# Differential Induction of the ADAM17 Regulators iRhom1 and 2 in Endothelial Cells

**DOI:** 10.3389/fcvm.2020.610344

**Published:** 2020-12-01

**Authors:** Aaron Babendreyer, Diana M. Rojas-González, Anja Adelina Giese, Sandra Fellendorf, Stefan Düsterhöft, Petra Mela, Andreas Ludwig

**Affiliations:** ^1^Institute of Molecular Pharmacology, University Hospital Rheinisch-Westfälische Technische Hochschule Aachen, Aachen, Germany; ^2^Department of Mechanical Engineering, Munich School of BioEngineering, Technical University of Munich, Garching, Germany

**Keywords:** ADAM, metalloprotease, iRhom1/2 (inactive Rhomboid like protein 1/2), endothelial cell, vascular inflammation

## Abstract

**Background:** Endothelial function significantly depends on the proteolytic release of surface expressed signal molecules, their receptors and adhesion molecules via the metalloproteinase ADAM17. The pseudoproteases iRhom1 and 2 independently function as adapter proteins for ADAM17 and are essential for the maturation, trafficking, and activity regulation of ADAM17. Bioinformatic data confirmed that immune cells predominantly express iRhom2 while endothelial cells preferentially express iRhom1.

**Objective:** Here, we investigate possible reasons for higher iRhom1 expression and potential inflammatory regulation of iRhom2 in endothelial cells and analyze the consequences for ADAM17 maturation and function.

**Methods:** Primary endothelial cells were cultured in absence and presence of flow with and without inflammatory cytokines (TNFα and INFγ). Regulation of iRhoms was studied by qPCR, involved signaling pathways were studied with transcriptional inhibitors and consequences were analyzed by assessment of ADAM17 maturation, surface expression and cleavage of the ADAM17 substrate junctional adhesion molecule JAM-A.

**Results:** Endothelial iRhom1 is profoundly upregulated by physiological shear stress. This is accompanied by a homeostatic phenotype driven by the transcription factor KLF2 which is, however, only partially responsible for regulation of iRhom1. By contrast, iRhom2 is most prominently upregulated by inflammatory cytokines. This correlates with an inflammatory phenotype driven by the transcription factors NFκB and AP-1 of which AP-1 is most relevant for iRhom2 regulation. Finally, shear stress exposure and inflammatory stimulation have independent and no synergistic effects on ADAM17 maturation, surface expression and JAM-A shedding.

**Conclusion:** Conditions of shear stress and inflammation differentially upregulate iRhom1 and 2 in primary endothelial cells which then results in independent regulation of ADAM17.

## Introduction

A functional endothelium is of immense importance for the organism, as it maintains the distribution of nutrients, gas exchange, vasotonus, but also ensures the recruitment of immune cells in the course of inflammation. The maintenance of these functions is influenced by mechanical forces, such as shear stress, among others. The blood flow exerts a constant shear stress on endothelial cells. Physiological levels of shear stress lead to a vasodilative, anti-thrombotic, anti-oxidative and anti-inflammatory phenotype in endothelial cells. This is transcriptionally controlled to a considerable extent either directly or indirectly by activation of the MEK5-MEF2-KLF2 (dual specificity mitogen-activated protein kinase kinase 5—myocyte enhancer factor-2—krüppel-like factor 2) cascade (1). If changes in the flow pattern occur, especially a permanent reduction of shear stress, the above mentioned effects are neutralized and endothelial dysfunction may occur (2). Endothelial dysfunction is associated with an inflammatory response of the endothelium (3). Inflammatory reactions may be beneficial to eliminate pathogens in the tissue or to initiate tissue repair. However, as mentioned above, chronic inflammation contributes to endothelial dysfunction, which can ultimately lead to cardiovascular diseases, such as atherosclerosis (4).

Limited proteolysis of membrane-anchored proteins at the cell surface can lead to the release of biologically active ectodomains. This type of cleavage is termed ectodomain shedding and plays an important role in several signaling pathways crucial for a variety of processes, such as the maintenance of endothelial homeostasis or inflammatory responses. In particular, the metalloproteinase ADAM17 from the family of a disintegrin and metalloproteinases (ADAM) is responsible for the shedding of more than 30 surface proteins in endothelial cells. These ADAM17 substrates include cytokines, such as tumor necrosis factor α (TNF) and chemokine (C-X3-C motif) ligand 1 (CX3CL1), cytokine receptors, such as TNF receptor 1 and 2 (TNFR1 and 2), growth factors, such as heparin-binding EGF-like growth factor (HB-EGF) and transforming growth factor α (TGFα), but also adhesion molecules, such as vascular cell adhesion molecule 1 (VCAM-1), junctional adhesion molecule A (JAM-A) and platelet endothelial cell adhesion molecule (PECAM-1) (5). This explains the importance of ADAM17 for the maintenance of endothelial function. Therefore, it is not surprising that ADAM17 is tightly regulated on several levels. One critical regulatory mechanism is the maturation of the protease. ADAM17 is synthesized with a pro-domain, which inhibits the catalytical domain of the protease. After ADAM17 has been transported from the endoplasmic reticulum to the Golgi apparatus, this pro-domain is cleaved by pro-protein convertases, such as furin. The proteolytically active, mature ADAM17 is transported to the cell surface (6). Two landmark studies in 2012 showed that iRhoms are critical regulators in this process as they are essential for the transport of ADAM17 from the endoplasmic reticulum into the Golgi apparatus (7, 8). iRhoms are catalytically inactive serine proteases from the rhomboid protease family. In mammals, two iRhoms are known, iRhom1 and 2. Whereas, iRhom1 is ubiquitously expressed in many cells and tissues, iRhom2 is believed to be expressed primarily in immune cells. However, for endothelial cells the expression and regulation of both iRhoms has not yet been investigated in detail.

In the present study, we found that both iRhoms are differentially regulated by shear stress and/or inflammatory cytokines on a transcriptional level in endothelial cells, which then influences the maturation, trafficking and function of ADAM17.

## Materials and Methods

### Antibodies, Recombinant Proteins, and Chemical Compounds

Mouse monoclonal antibody against the mature form of human ADAM17 (clone 111633) and mouse IgG1 isotype control were from R&D Systems (Wiesbaden, Germany). Rabbit polyclonal antibody against human ADAM17 was from Chemicon (Darmstadt, Germany). Allophycocyanin (APC)-conjugated goat anti-mouse antibody and horse radish peroxidase (HRP)-conjugated goat anti-mouse were from Jackson ImmunoResearch Laboratories, Inc. (West Grove, USA). Tumor necrosis factor α (TNFα) and interferon γ (IFNγ) were from PreproTech (Rocky Hill, USA). Simvastatin was from Merck KGaA (Darmstadt, Germany). Geranylgeranyl pyrophosphate (GGPP) and the MEK5 inhibitor BIX02189 were from Sigma-Aldrich (St. Louis, USA). Activator protein 1 (AP-1) inhibitor SR11302 was from Tocris Bioscience (Bristol, UK). Nuclear factor kappa-light-chain-enhancer of activated B cells (NF-κB) inhibitor Bay11-7082 was from Santa Cruz Biotechnology (Dallas, USA).

### Bioinformatic Analysis of Public Transcriptomic Data Sets

We performed a bioinformatic analysis of mRNA expression data of human samples generated by Affymetrix Human Genome U133Plus 2.0 Arrays from a variety of public repositories with the tool Genevestigator v8 suite (Nebion AG, Zürich, Switzerland) (9).

### Cell Culture

Human pulmonary microvascular endothelial cells (HPMECs) were from Promocell (Heidelberg, Germany). Human adipose microvascular endothelial cells (HAMECs) were from ScienceCell (Carlsbad, USA). Human umbilical vein endothelial cells (HUVECs) and human umbilical artery endothelial cells (HUAECs) were isolated from the umbilical cord of cesarean sections in our laboratory as described (10) and cultured in Endopan-3 from PAN-Biotech (Aidenbach, Germany). This work was approved by the local ethic committee of the medical faculty RWTH Aachen with the ethical vote EK241/18. For a better comparison all endothelial cells were cultured in endothelial cell growth medium MV2 (Promocell). Each experiment with either HUVECs or HUAECs was performed with cells that were prepared from a different donor and each experiment with either HPMECs or HAMECs was performed with cells from two different donors.

### Flow Experiments

For flow experiments, cells were used in passage four to six and seeded with a density of 40,000 cells/cm^2^ in ibidi μ-Slides of different type (0.2 and 0.8 mm μ-slides, Martinsried, Germany) or a 24-well plate for the static control. The flow in the μ-Slides was accomplished with the ibidi pump system. Depending on the geometry of the μ-slide type, the flow rate was adjusted as specified by the manufacturer to yield the desired laminar shear stress. Flow experiments were started 2.5 h after seeding and performed for 24 h. Since only one level of shear stress could be applied, experiments with different levels of shear stress had to be performed on different days. Therefore, a static control was performed for each level of shear stress. However, all static controls were included in one column for a clearer presentation of the data. For MEK5 inhibition, 10 μM BIX021879 was added to the cells directly before seeding and then cells were cultured for 24 h under flow conditions with growth medium containing 10 μM BIX02189. In case of flow experiments with additional TNFα treatment, cells were first cultured for 24 h without 10 ng/ml TNFα and then for additional 24 h with TNFα, which means a total flow cultivation of 48 h.

### Simvastatin Treatment

For KLF2 induction, HUVECs were treated for 24 h with 1 μM simvastatin. To suppress the simvastatin mediated KLF2 induction, treatment of HUVECs with 1 μM simvastatin was performed in the presence of 10 μM GGPP for 24 h.

### siRNA Transfection

Directly before transfection, HUVECs were seeded in a 24-well plate with a density of 3.5 × 10^4^ cells/well in growth medium. Subsequently, transfection solution of 50 μl Opti-MEM® reduced serum medium (Thermo Fisher Scientific, Waltham, USA), 1 μl Lipofectamin™ RNAiMAX (Invitrogen, Carlsbad, USA) and 30 nM small interfering RNA (siRNA) targeting human KLF2 (stealth RNAi™ HSS145585) or unspecific control siRNA (Invitrogen) was added. After 24 h, the medium was replaced and transfected cells were used for stimulation and flow experiments.

### Cytokine Treatment

For cytokine treatment cells were used in passage four to six and seeded with a density of 100,000 cells/cm^2^ in a 24-well plate and cultured for 24 h with indicated concentrations of TNFα and/or IFNγ. For AP-1 and NF-κB inhibition, cells were cultured and pretreated for 1 h with 10 μM SR11302 or 3 μM Bay11-7082, respectively.

### RT-qPCR

The mRNA levels were quantified by RT-qPCR analysis and normalized to the mRNA level of different reference genes. The most stable reference genes were determined with the geNorm algorithm included in the qbase+ software (biogazelle, Gent, Belgium). Based on these results, glyceraldehyde 3-phosphate dehydrogenase (GAPDH) and TATA-binding protein (TBP) were chosen as most stable reference genes. Since normalization was always performed against these two reference genes, we have introduced the term reference gene index (ref. index) to make the y-scale clearer. RNA was extracted using RNeasy Kit (Qiagen, Hilden, Germany) and quantified photometrically. For each set of experiments equal amounts of RNA were reverse transcribed using PrimeScript™ RT Reagent Kit (Takara Bio Europe, St-Germain-en-Laye, France) and PCR reactions were performed using iTaq Universal SYBR Green Supermix (Bio-Rad, Hercules, USA) according to the manufacturer's protocol. The specific primers and annealing temperatures are listed in Supplementary Table 1. All PCR reactions were run on a CFX Connect Real-Time PCR Detection System (Bio-Rad) with the following protocol: 40 cycles of 10 s denaturation at 95°C, followed by 10 s annealing at the indicated temperature and 15 s amplification at 72°C. PCR efficiency was determined from the uncorrected RFU values using LinRegPCR version 2020.0 (11). Relative quantification was performed with the CFX Maestro Software 1.1 (Bio-Rad).

### Western Blotting

Cultured HUVECs were resuspended at 1 × 10^6^ cells per ml in lysis buffer (20 mM Tris-HCl, 150 mM NaCl, 1% TritonX-100, 1 mM EDTA, 1 mM Na_3_VO_4_, 1 mM PMSF, 10 mM 1,10-phenanthroline monohydrate) supplemented with Complete Inhibitor (Roche) and SDS sample buffer (with final concentrations of 2% SDS, 50 mM Tris-HCl, 10% glycerol, and 0.02% bromophenol blue) and incubated for 10 min. After centrifugation at 16,000 g for 5 min, supernatants were investigated by reducing SDS-PAGE. Proteins were transferred onto polyvinylidene difluoride membranes (Hybond-P, Amersham) and probed with primary polyclonal antibody against ADAM17 (0.1 μg/ml) or monoclonal antibody against GAPDH (1 μg/ml) over night at 4°C followed by HRP-coupled polyclonal secondary antibody (30 ng/ml in PBS-T with 2% non-fat dry milk) for 1 h. After addition of enhanced chemiluminescence substrate (ECL, Thermo Fisher Scientific, Waltham, USA), signals were recorded and quantified using the LAS 3000 *Image Analyzer*^®^ (Fujifilm, Tokyo, Japan). ADAM17 was detected as two protein bands representing the mature form of ADAM17 (mADAM17) and the pro form of ADAM17 (pADAM17) of ~100 and 130 kDa, respectively. Densitometric analysis was performed with Image Studio Lite (Li-Cor, Lincoln, NB, USA). ADAM17 total protein (tADAM17) was determined as signal density of pADAM17 and mADAM17 together. Protein levels were then expressed as signal density ratio of either tADAM17 and GAPDH or pADAM17 and mADAM17.

### ELISA Measurements

The release of human JAM-A into the supernatant was analyzed by ELISA. Before the measurement, the culture supernatants were concentrated from 3 to 0.5 ml using Vivaspin 6 columns (10,000 MWCO) (Sartorius, Göttingen, Germany). The ELISA was performed according to manufacturer's instructions (JAM-A ELISA kit, SinoBiological, Beijing, China). The chromogenic reaction was mediated by a standard procedure using 0.1 U/ml streptavidin-conjugated horseradish peroxidase (Roche, Basel, Switzerland) and the BM Blue POD substrate (Roche).

### Flow Cytometric Analysis

PBS supplemented with 0.2% BSA was used as assay buffer, and all steps of the staining process were performed at 4°C. HUVECs were analyzed for expression of ADAM17 by incubation with a mouse monoclonal antibody against ADAM17 (2 μg/ml) followed by incubation with APC-conjugated anti-mouse antibody (1:200). An IgG1 isotype control was used in parallel. The fluorescence signal was detected by flow cytometry (LRS Fortessa, BD Biosciences) and analyzed with FlowJo 8.7.3 software (Tree Star, Inc., Ashland, USA).

### Statistics

Quantitative data are shown as mean and standard deviation (SD) calculated from at least three independent experiments. Statistics were conducted using the general mixed model analysis (PROC GLIMMIX, SAS 9.4, SAS Institute Inc., Cary, USA). Data were analyzed for the optimal distribution, using Akaike Information Criterion (AIC), the Bayesian Information Criterion (BIC), residual plots and the Shapiro-Wilk test as diagnostics. If necessary, the donor was set as random term to assess for donor-specific differences. In the case of heteroscedasticity (according to the covtest statement) the degrees of freedom were adjusted by the Kenward-Roger approximation. If the data still did not represent a normal distribution (according to the Shapiro-Wilk test), a non-parametric Kruskal-Wallis test was performed (GraphPad Prism 7, GraphPad Software, San Diego, USA). Multiple comparisons were corrected by false discovery rate (FDR). A *p*-value < 0.05 was considered significant.

## Results

### Bioinformatic Analysis of Public Transcriptomic Data

We used bioinformatic analysis of transcriptome data from human samples generated with Affymetrix Human Genome U133Plus 2.0 Arrays to obtain an overview of the mRNA expression levels of iRhom1 and iRhom2 in different tissues and cell types. For this purpose, array data from different public repositories were analyzed using the Genevestigator suite (9). We investigated expression patterns of iRhom1 and iRhom2 in the data sets of untreated, mock or placebo treated samples of human origin. We found that the majority of tissues and cell types analyzed express both iRhom1 and iRhom2. However, the ratio of expression levels of iRhom1 and iRhom2 was often very different (Supplementary Figure 1). As expected, the expression of iRhom2 was significantly higher in immune cells than that of iRhom1. Strikingly, in endothelial cells this was the opposite. These cells showed the highest expression level of iRhom1. Therefore, we decided to investigate which conditions could drive the expression of iRhom1 in endothelial cells. First, we chose shear stress as physiological stimulus, which plays an important role in the physiology of endothelial cells. Since iRhom2 has already been shown to be relevant for inflammatory processes in various tissues, we also investigated the influence of inflammatory cytokines on iRhom2 expression in the later course of the study.

### Shear Stress Induces Endothelial iRhom mRNA Expression

**Figure 1:**
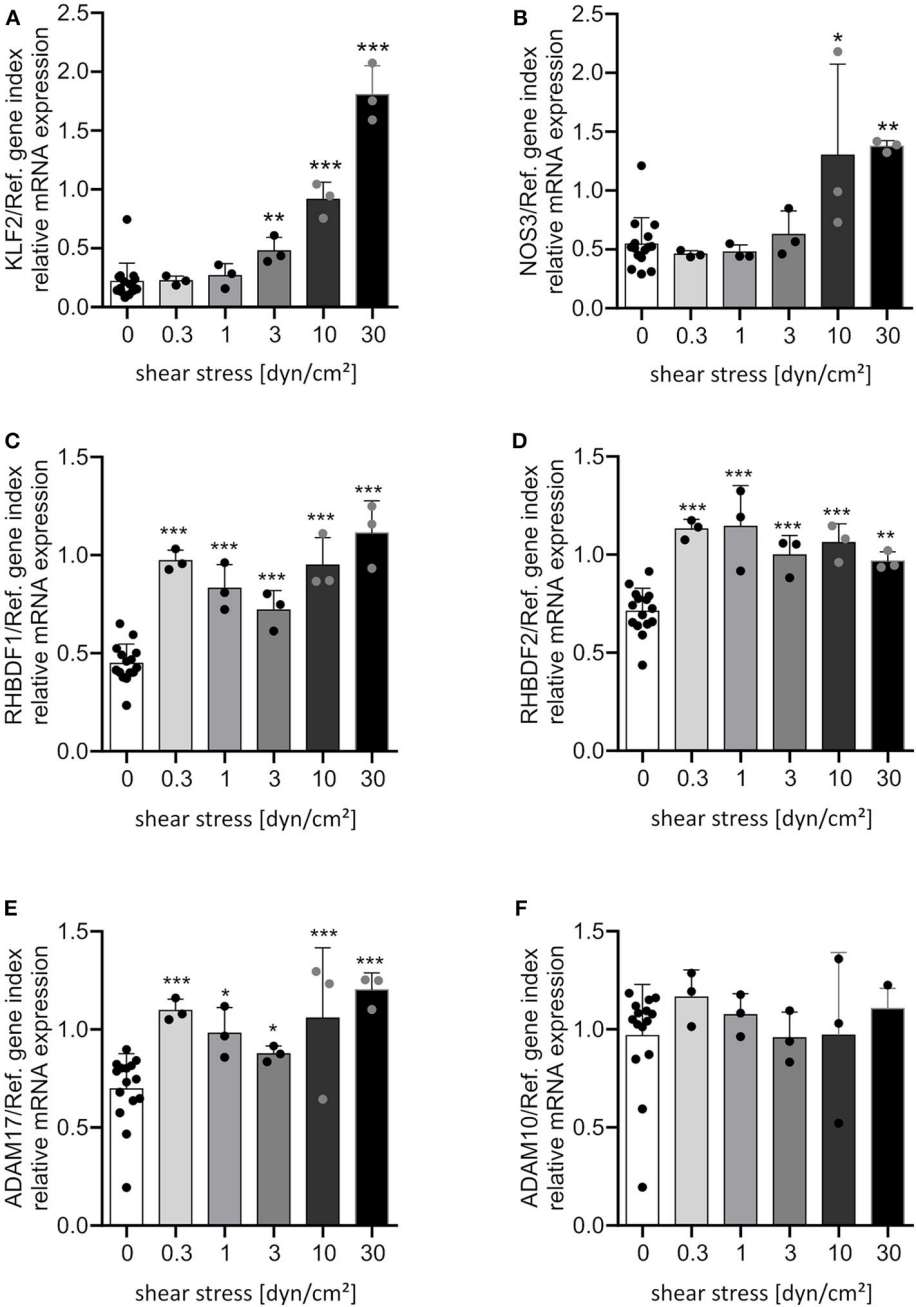
Shear stress induces most prominently iRhom1. HUVECs were cultured for 24 h under different flow conditions resulting in the indicated levels of laminar shear stress. Cells were then analyzed for mRNA expression of KLF2 **(A)**, NOS3 **(B)**, RHBDF1 **(C)**, RHBDF2 **(D)**, ADAM17 **(E)**, and ADAM10 **(F)** in relation to a reference gene index consisting of GAPDH and TBP. The three independent experiments were performed with HUVECs from three different donors. A static control (0 dyn/cm^2^) was made for each shear stress level. All static controls were merged in one column for clarity. Data are shown as mean + standard deviation (SD) and as black and gray dots representing the individual data points. Statistical differences to the static control are indicated by asterisks (*p < 0.05, **p < 0.01, and ***p < 0.001).

To investigate the effect of shear stress, human umbilical vein endothelial cells (HUVECs) were cultured under static or different flow conditions. Characteristic for endothelial cells cultivated under flow is the transcriptional induction of the transcription factor KLF2. KLF2 is then directly or indirectly responsible for the transcriptional regulation of the majority of shear stress sensitive genes (12). Therefore, we controlled the mRNA expression of KLF2 and two of its target genes to verify the quality of our flow experiments. As expected, increasing levels of shear stress led to an increasing mRNA expression of the transcription factor KLF2 in a dose dependent manner (Figure 1A). Along with this, mRNA expression of the direct KLF2 target gene encoding the endothelial nitric oxide synthase (*NOS3*) was upregulated (Figure 1B) whereas the mRNA expression of the indirect target gene encoding endothelin-1 (*EDN1*) was downregulated (Supplementary Figure 2). These and other transcriptional KLF2-dependent regulations lead to the typical shear stress-induced protective phenotype. While KLF2 induction requires 3 dyn/cm^2^ of shear stress and gradually increases with higher shear stress (Figure 1A), the mRNA expression of the iRhom1 and 2 genes *RHBDF1* and *RHBDF2* were induced already at 0.3 dyn/cm^2^ and this response was not further increased by higher shear stress (Figures 1C,D). The induction of iRhom1 was with an average 2-fold increase more prominent than that of iRhom2 (1.5-fold). Additionally, ADAM17 showed a slight but significant transcriptional increase after flow cultivation at all tested shear stress levels (Figure 1E). For comparison, we analyzed mRNA expression of ADAM10, but no influence of flow cultivation was observed (Figure 1F).

**Figure 2:**
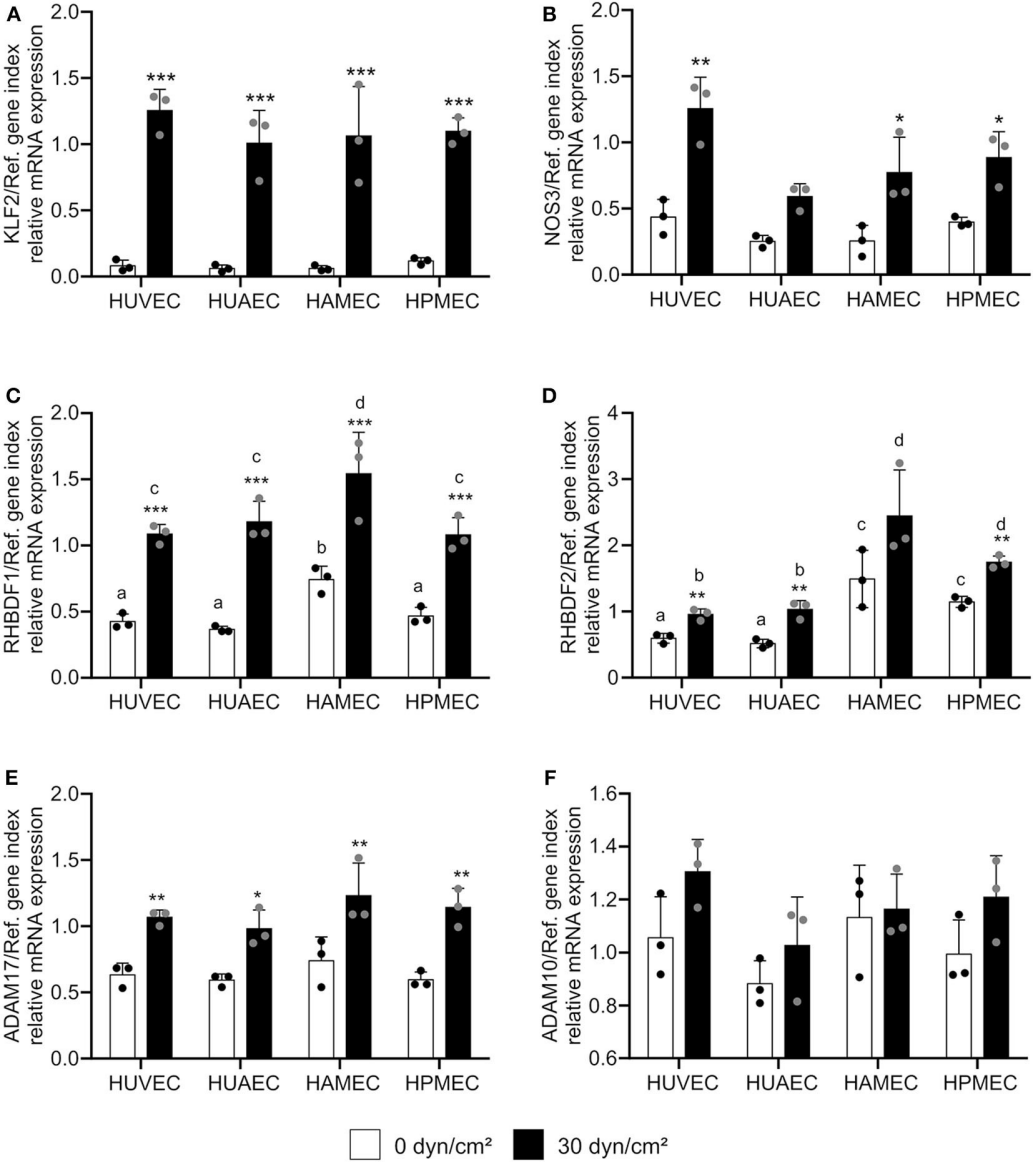
High physiological shear stress induces most prominently iRhom1 in endothelial cells from different vascular beds. HUVECs, HUAECs, HAMECs, and HPMECs were cultured for 24 h with a laminar shear stress of 30 dyn/cm^2^. Cells were then analyzed for mRNA expression of KLF2 **(A)**, NOS3 **(B)**, RHBDF1 **(C)**, RHBDF2 **(D)**, ADAM17 **(E)**, and ADAM10 **(F)** in relation to a reference gene index consisting of GAPDH and TBP. The three independent experiments were performed with HUVECs and HUAECs from three different donors and HAMECs and HPMECs from two different donors. Data are shown as mean + standard deviation (SD) and as black and gray dots representing the individual data points. Statistical differences to the static control are indicated by asterisks (*p < 0.05, **p < 0.01, and ***p < 0.001). Significant differences between the different endothelial cell types were marked with different letters (a, b, c, and d). Each group with one letter is significantly different from a group with another letter.

Next, we questioned whether this transcriptional regulation could be reproduced in endothelial cells from different vascular beds. In addition to HUVECs, human umbilical artery endothelial cells (HUAECs), human adipose microvascular endothelial cells (HAMECs) and human pulmonary microvascular endothelial cells (HPMECs) were cultivated under static and flow conditions. We decided to use a shear stress of 30 dyn/cm^2^, because this was a condition with strongest effects on KLF2-mediated transcriptional regulation (Figures 1A,B; Supplementary Figure 2) and more importantly because it is in a physiological range for microvascular endothelial cells (13). All endothelial cell types showed a comparable response to shear stress at 30 dynes/cm^2^ after 24 h with respect to the shear stress-mediated transcriptional regulation of KLF2 and its target genes (Figures 2A,B, Supplementary Figure 3). Importantly, the strong inductive effect of shear stress on iRhom1 was confirmed with a 2-fold or even higher increase in all endothelial cell types (Figure 2C). In contrast, mRNA expression of iRhom2 and ADAM17 again showed a weaker induction in the range of a 1.5-fold increase (Figures 2D,E) and ADAM10 was not transcriptionally regulated (Figure 2F). Interestingly, this experiment also revealed that HAMECs constitutively express significantly more iRhom1 than endothelial cells derived from the umbilical cord (Figure 2C). In the case of iRhom2 even both microvascular cell types showed an increased basal expression compared to umbilical cord cells (Figure 2D).

In conclusion, shear stress induces iRhom mRNA expression in endothelial cells from different vascular beds, with the induction of iRhom1 being much stronger compared to the induction of iRhom2.

### Shear Stress-Mediated iRhom1 Induction Only Partially Depends on KLF2 Pathway

**Figure 3:**
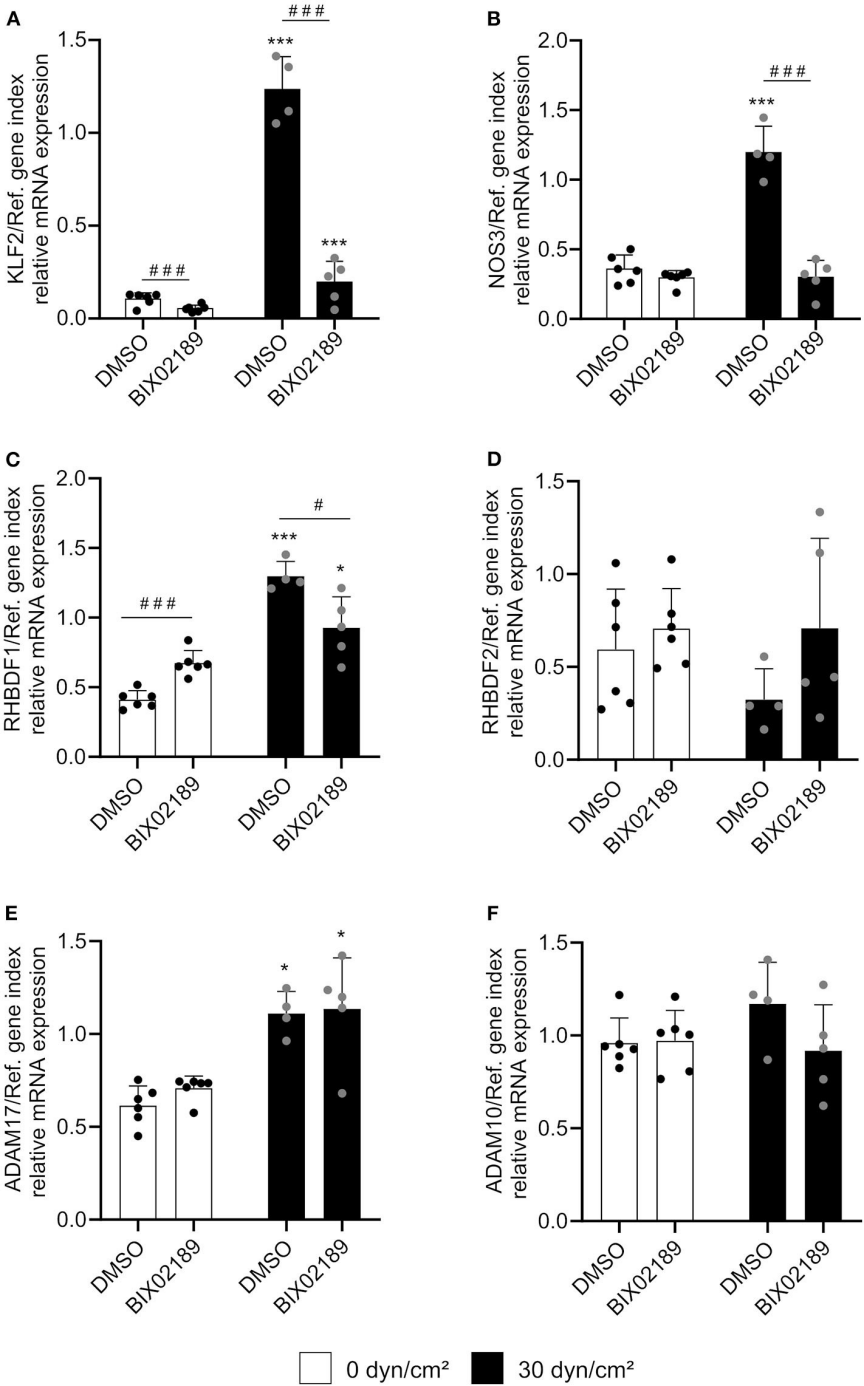
Shear stress-mediated iRhom1 mRNA expression is partially suppressed by MEK5 inhibition. HUVECs were pretreated with DMSO or 10 μM of the MEK5 inhibitor BIX02189 for 2.5 h and cultured for 24 h with a laminar shear stress of 30 dyn/cm^2^ in the presence of DMSO or BIX02189. Cells were then analyzed for mRNA expression of KLF2 **(A)**, NOS3 **(B)**, RHBDF1 **(C)**, RHBDF2 **(D)**, ADAM17 **(E)**, and ADAM10 **(F)** in relation to a reference gene index consisting of GAPDH and TBP. At least four independent experiments were performed with HUVECs from four different donors. Data are shown as mean + standard deviation (SD) and as black and gray dots representing the individual data points. Statistical differences to the corresponding static control are indicated by asterisks (*p < 0.05, **p < 0.01, and ***p < 0.001) and significant differences between DMSO and the MEK5 inhibitor are indicated as hashes (^#^*p* < 0.05, ^##^*p* < 0.01, and ^###^*p* < 0.001).

The next step was to investigate the influence of the KLF2 signaling pathway on the shear stress-mediated iRhom mRNA induction. For this purpose, HUVECs were treated with 10 μM of the specific MEK5 inhibitor BIX02819 before and during the flow experiment for 24 h. MEK5 lies upstream of the KLF2 signaling pathway (14). As expected, the inhibitor suppressed the shear stress-mediated transcriptional induction of KLF2 and NOS3 mRNA expression (Figures 3A,B). However, the shear stress-mediated transcriptional inhibition of the indirect KLF2 target gene *EDN1* could not be reversed (Supplementary Figure 4). This can be explained by the fact that anti-inflammatory effects can also be directly mediated by shear stress-dependent eNOS activation and subsequent NO production (15).

**Figure 4:**
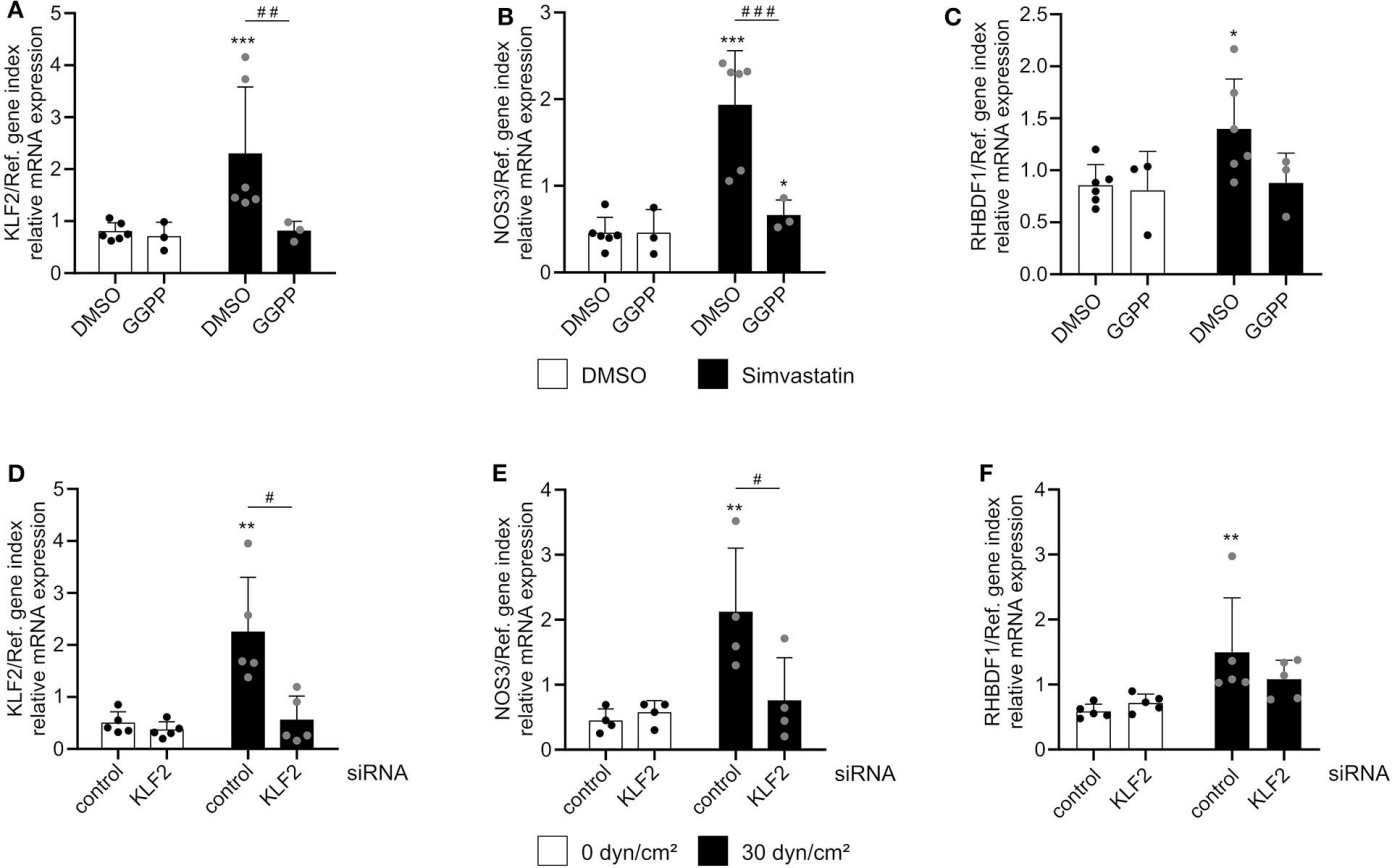
Regulation of iRhom1 mRNA expression by interference with the KLF2 pathway. **(A–C)** HUVECs were treated for 24 h with 1 μM simvastatin or control vehicle (DMSO) in the presence or absence of 10 μM GGPP. Cells were then analyzed for mRNA expression of KLF2 **(A)**, NOS3 **(B)**, and RHBDF1 **(C)**. **(D–F)** HUVECs were transfected with non-targeting control siRNA or siRNA against KLF2. After 24 h, cells were cultured under static conditions or with a shear stress of 30 dyn/cm^2^ for 24 h and analyzed for their mRNA expression of KLF2 **(D)**, NOS3 **(E)**, and RHBDF1 **(F)**. At least three **(A–C)** or four **(D–F)** independent experiments were performed with HUVECs from at least three or four different donors. Data are shown as mean + standard deviation (SD) and as black and gray dots representing the individual data points. Statistical differences to the corresponding static control are indicated by asterisks (*p < 0.05, **p < 0.01, and ***p < 0.001) and significant differences between DMSO and GGPP or control siRNA and siRNA against KLF2 are indicated as hashes (^#^*p* < 0.05, ^##^*p* < 0.01, and ^###^*p* < 0.001).

The shear stress-mediated induction of iRhom1 was significantly reduced upon MEK5 inhibition. However, the inhibitor also led to a significant increase of basal iRhom1 expression under static conditions (Figure 3C). Due to this shear stress-independent effect of the inhibitor it cannot be clearly evaluated to what extent MEK5 contributes to the induction of iRhom1. For the other genes investigated, the inhibitor did not show significant effects on the mRNA expression (Figures 3D–F). To further study the potential influence of KLF2, its expression was pharmacologically induced in HUVECs by inhibition of HMG-CoA reductase via simvastatin treatment. This effect can be reversed by adding the metabolite geranylgeranyl pyrophosphate, which is produced downstream of the HMG-CoA reductase (16). As expected, a simvastatin stimulation for 24 h showed an induction of KLF2 and NOS3 expression, which could be reversed by the addition of GGPP (Figures 4A,B). Noteworthy, we also observed an induction of iRhom1 expression (Figure 4C) which was, however, lower than under shear stress (Figure 1C). Finally, siRNA was used to knockdown KLF2 expression in HUVECs and the effect on flow culture was investigated. As expected, the KLF2 knockdown suppressed the shear stress-dependent KLF2 and NOS3 induction (Figures 4D,E). In KLF2 knockdown cells, we could not observe a significant induction of iRhom1 by shear stress. However, due to high variations of iRhom induction in flow cultured cells no clear significant effect between control cells and cells with the KLF2 knockdown could be observed (Figure 4F). To illustrate this, we have also plotted the data normalized against the corresponding static control (Supplementary Figure 5).

Taken together, these different experiments indicate, a partial involvement of the MEK5-KLF2 pathway in iRhom1 but not iRhom2 induction. However, this does not seem to be the only mechanism contributing to the transcriptional regulation of iRhom1 by shear stress.

### TNFα Is a Potent Inducer of iRhom2 mRNA Expression

**Figure 5:**
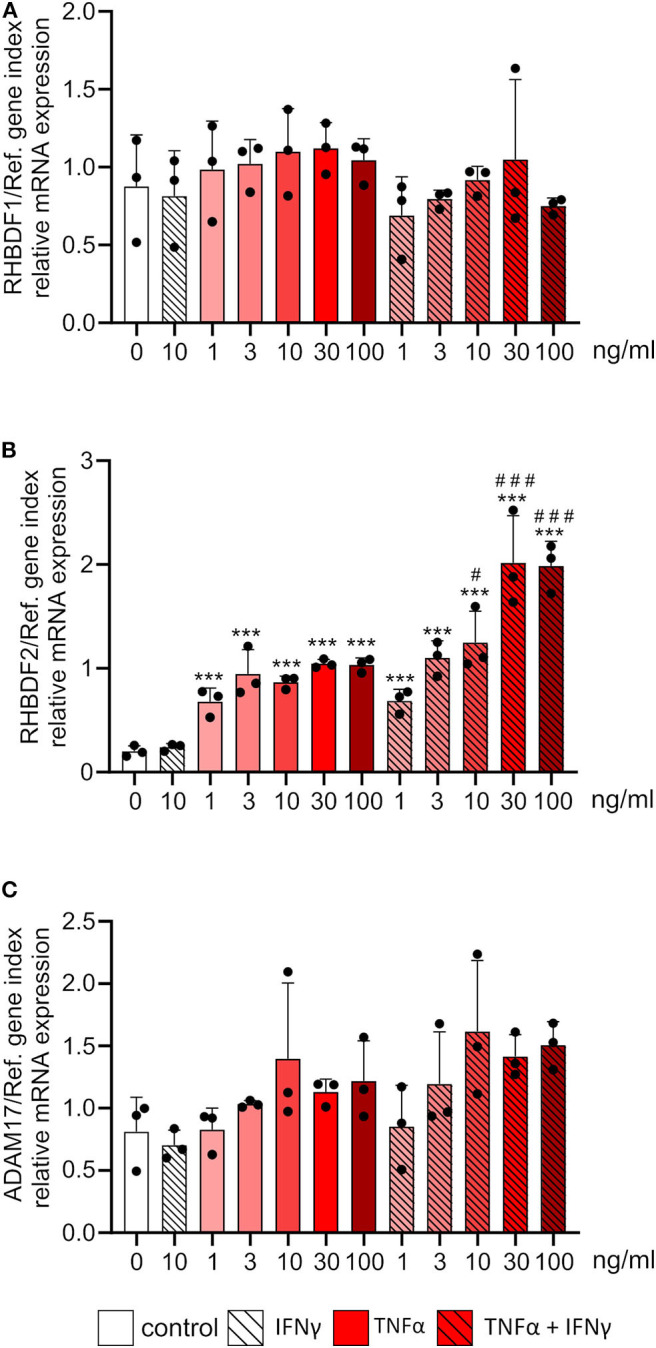
Inflammatory cytokines exclusively induce the mRNA expression of iRhom2 in endothelial cells. HUVECs were treated for 24 h with the indicated concentrations of TNFα, IFNγ or the combination of TNFα and IFNγ. Cells were then analyzed for mRNA expression of RHBDF1 **(A)**, RHBDF2 **(B)**, and ADAM17 **(C)**. Three independent experiments were performed with HUVECs from three different donors. Data are shown as mean + standard deviation (SD) and as black and gray dots representing the individual data points. Statistical differences to the untreated control cells are indicated by asterisks (*p < 0.05, **p < 0.01, and ***p < 0.001) and significant differences between TNFα and IFNγ treated cells to the corresponding cells treated with TNFα alone are indicated as hashes (^#^*p* < 0.05, ^##^*p* < 0.01, and ^###^*p* < 0.001).

Next, we investigated the effect of the inflammatory mediators, TNFα and IFNγ, on the mRNA expression of iRhoms. HUVECs were treated with different concentrations of TNFα or the combination of TNFα and IFNγ for 24 h. While there was no effect of TNFα or the combination of TNFα and IFNγ on iRhom1 mRNA expression, iRhom2 mRNA expression was strongly induced (Figures 5A,B). The treatment of TNFα alone resulted in a 5-fold increase in expression even at moderate concentrations. The combination of TNFα and IFNγ in high concentrations even led to a 10-fold increase in mRNA expression. Thus, to some extent, a synergistic effect of high TNFα and IFNγ concentrations on the expression of iRhom2 could be observed. As a control gene for successful cytokine stimulation we analyzed the expression of the chemokine CX3CL1, which is known to be induced by TNFα and IFNγ in a synergistic manner (17). Here the synergistic effect of the combination of both cytokines led to a 10-fold increase compared to the stimulation with TNFα alone. Notably, this synergistic effect could already be observed at low cytokine concentrations and the synergistic induction of CX3CL1was clearly stronger than that observed for iRhom2 (Supplementary Figure 6). For ADAM17 expression, no significant effect of TNFα and the combination of TNFα and IFNγ was observed, but there was a slight tendency toward an induction (Figure 5C).

### TNFα Mediated iRhom2 Induction Is Predominantly Mediated by AP-1

**Figure 6:**
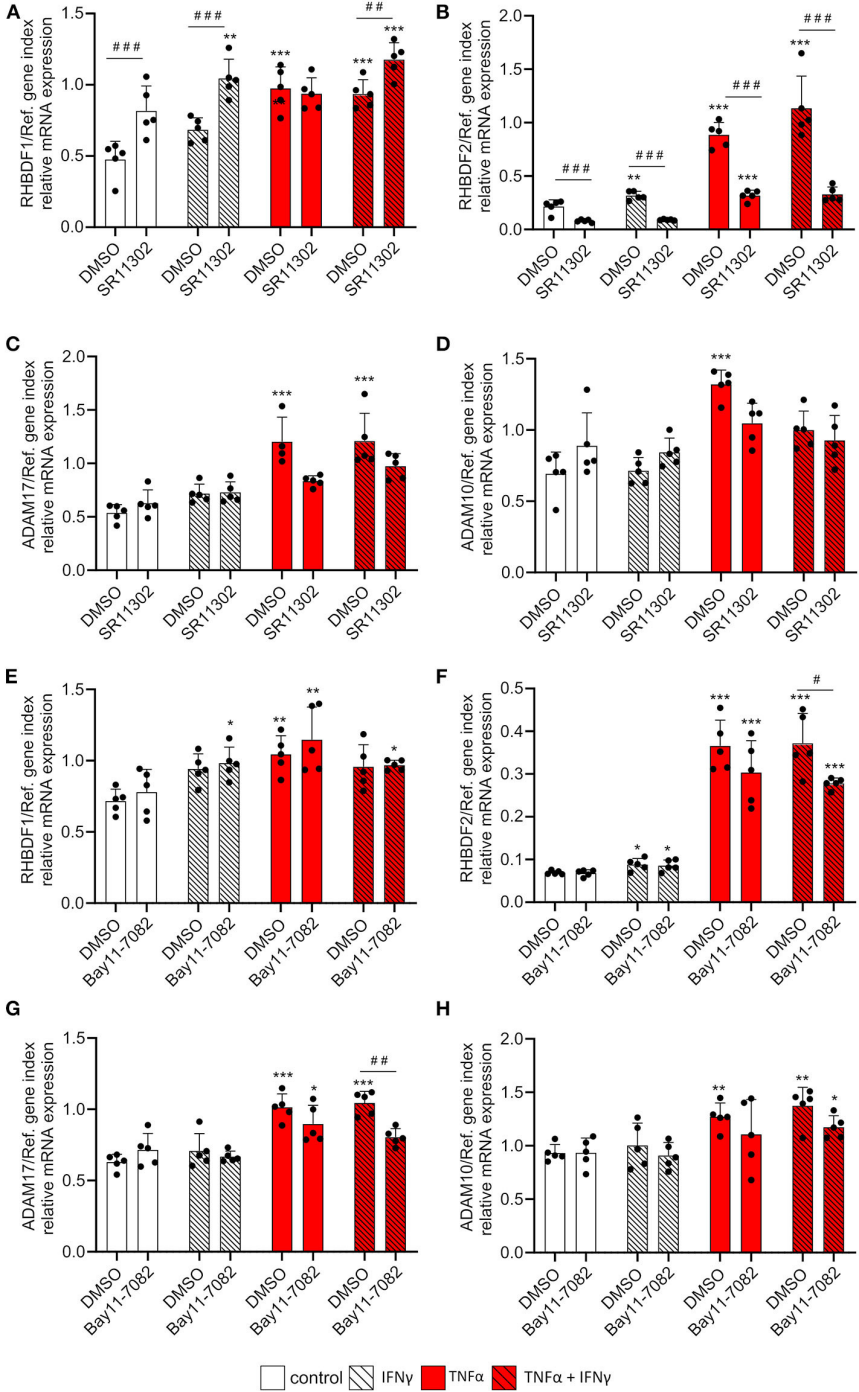
TNFα-induced iRhom2 expression is mainly controlled by AP-1 in endothelial cells. HUVECs were pretreated with DMSO, 10 μM of the AP-1 inhibitor SR11302 **(A–D)** or 3 μM of the NFκB inhibitor Bay11-7082 **(E–H)** for 1 h and cultured for 24 h with 10 ng/ml TNFα, IFNγ or the combination of TNFα and IFNγ in the presence of DMSO, SR11302, or Bay11-7082. Cells were then analyzed for mRNA expression of RHBDF1 **(A,E)**, RHBDF2 **(B,F)**, ADAM17 **(C,G)**, and ADAM10 **(D,H)**. Five independent experiments were performed with HUVECs from five different donors. Data are shown as mean + standard deviation (SD) and as black and gray dots representing the individual data points. Statistical differences to the corresponding untreated control cells are indicated by asterisks (*p < 0.05, **p < 0.01, and ***p < 0.001) and significant differences between DMSO and SR11302 or Bay11-7082 are indicated as hashes (^#^p < 0.05, ^##^*p* < 0.01, and ^###^*p* < 0.001).

We then investigated the influence of the inflammatory transcription factors AP-1 and NFκB, which are the main transcription factors involved in TNFα-mediated transcriptional regulation, on the cytokine-mediated iRhom2 induction. To this end, HUVECs were stimulated for 24 h with 10 ng/ml TNFα, IFNγ or the combination of both in the absence or presence of 10 μM of the AP-1 inhibitor SR11302 and 3 μM of the NFκB inhibitor Bay11-7082. We chose a cytokine concentration of 10 ng/ml to exclude effects of TNF-induced apoptosis on the mRNA expression of iRhoms and ADAMs. Both inhibitors showed a clear, and in most cases complete inhibition of cytokine-induced CX3CL1 mRNA expression (Supplementary Figures 7A,B). As explained above, TNFα and IFNγ had only marginal effects on mRNA expression of iRhom1 and these were not reduced by either the AP-1 or NFκB inhibitor (Figures 6A,E). The AP-1 inhibitor even showed a significant induction of iRhom1 expression. By contrast, iRhom2 mRNA expression was considerably induced by TNFα and IFNγ and this response was clearly reduced by the inhibition of AP-1 (Figure 6B). Of note, the AP-1 inhibitor also reduced the basal iRhom2 expression in unstimulated cells. In comparison, the NFκB inhibitor had only a small reducing effect on the cytokine-induced iRhom2 mRNA expression and no effect on the basal expression (Figure 6F). The AP1 inhibitor and the NFκB inhibitor also showed a small reduction in ADAM17 mRNA expression induced by the combination of TNFα and IFNγ but this effect was only significant for the NFκB inhibitor (Figure 6G). The mRNA expression levels of ADAM10 remained unaffected by both inhibitors (Figures 6C,D,G,H).

In summary, our data indicate that TNFα is a strong inducer of iRhom2 mRNA expression in endothelial cells and this induction is mainly mediated by the transcription factor AP-1.

### Shear Stress and TNFα Independently Induce mRNA Expression of Both iRhoms

Previous studies have revealed that transcriptional pathways induced by flow or by inflammatory cytokines can interfere with each other (14). We therefore questioned how iRhom mRNA expression is affected when cells are simultaneously treated with shear stress and TNFα, and how this influences ADAM17 maturation and shedding of ADAM17 substrates. For this purpose, HUVEC were first incubated with or without flow at 30 dyn/cm^2^ for 24 h and then stimulated with 10 ng/ml TNFα in the presence or absence of flow for another 24 h. First, we controlled how TNFα stimulation would affect flow induced signaling pathways. Although shear stress-mediated KLF2 induction was not influenced by additional TNFα treatment (Supplementary Figure 8A), shear stress-induced NOS3 mRNA expression was significantly reduced by TNFα (Supplementary Figure 8B).

**Figure 7:**
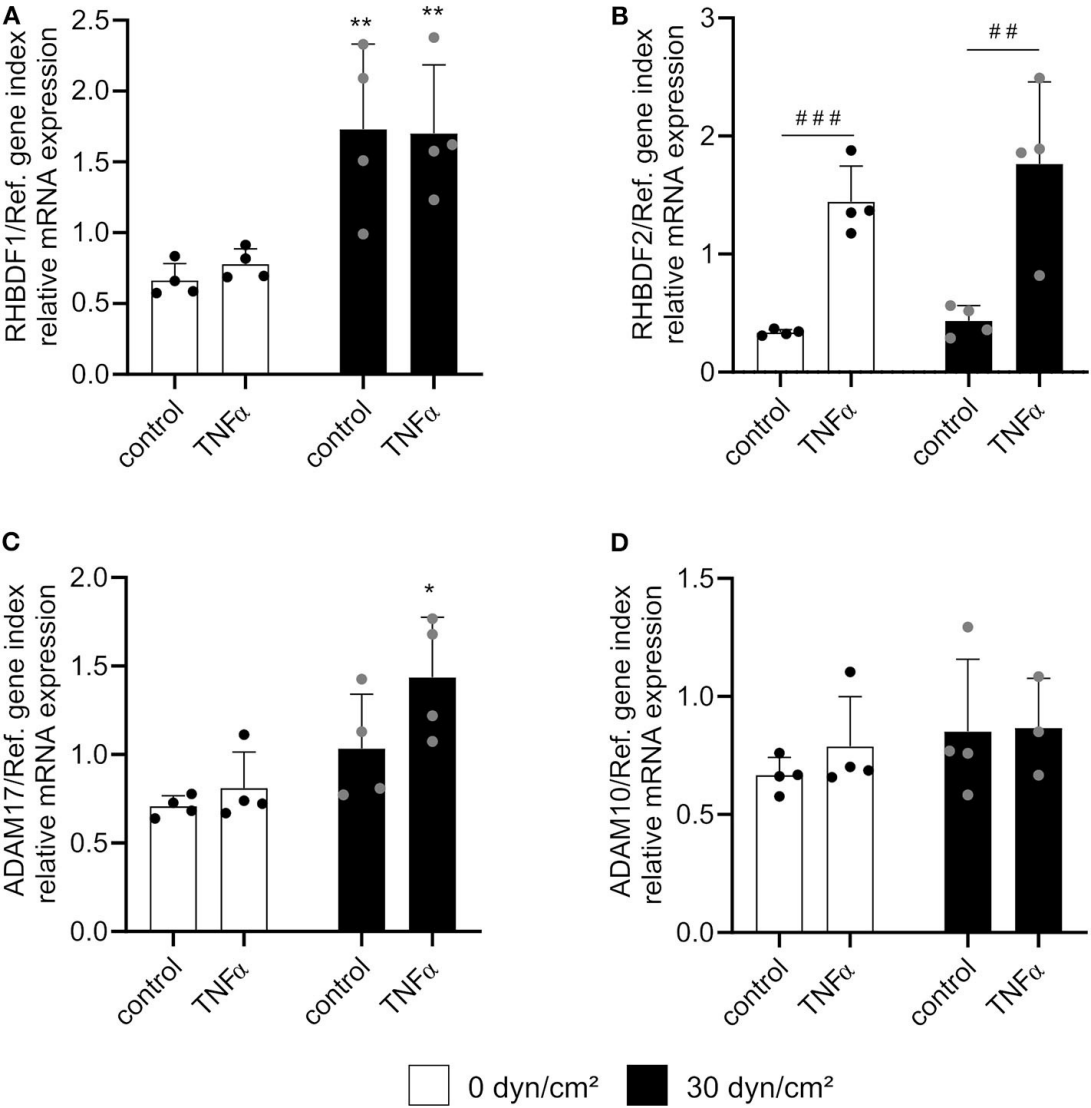
Shear stress and TNFα induce iRhom1 or iRhom2 in an independent manner. HUVECs were cultured for 24 h under static conditions or with a shear stress of 30 dyn/cm^2^ and subsequently stimulated with or without 10 ng/ml TNFα for another 24 h with or without flow. Cells were then analyzed for mRNA expression of RHBDF1 **(A)**, RHBDF2 **(B)**, ADAM17 **(C)**, and ADAM10 **(D)**. Four independent experiments were performed with HUVECs from four different donors. Data are shown as mean + standard deviation (SD) and as black and gray dots representing the individual data points. Statistical differences to the corresponding static control are indicated by asterisks (*p < 0.05, **p < 0.01, and ***p < 0.001) and significant differences between untreated control cells and cells treated with TNFα are indicated as hashes (^#^*p* < 0.05, ^##^*p* < 0.01, and ^###^*p* < 0.001).

As expected, TNFα treatment alone selectively induced iRhom2 while flow exposure predominantly induced iRhom1 mRNA expression. Interestingly, no significant change of mRNA expression of both iRhoms could be observed between the combination of flow cultivation and TNFα stimulation and the single treatments (Figures 7A,B). Importantly, there was no negative effect on the respective treatments, as it was observed for the mRNA expression of the *NOS3* gene (Supplementary Figure 8B). Interestingly, a slight synergistic effect of TNFα and flow exposure on ADAM17 mRNA expression was observed (Figure 7C). The expression level of ADAM10 mRNA was not affected by this combination, as expected (Figure 7D).

### Shear Stress and TNFα Stimulation Independently Enhance ADAM17 Surface Expression

**Figure 8:**
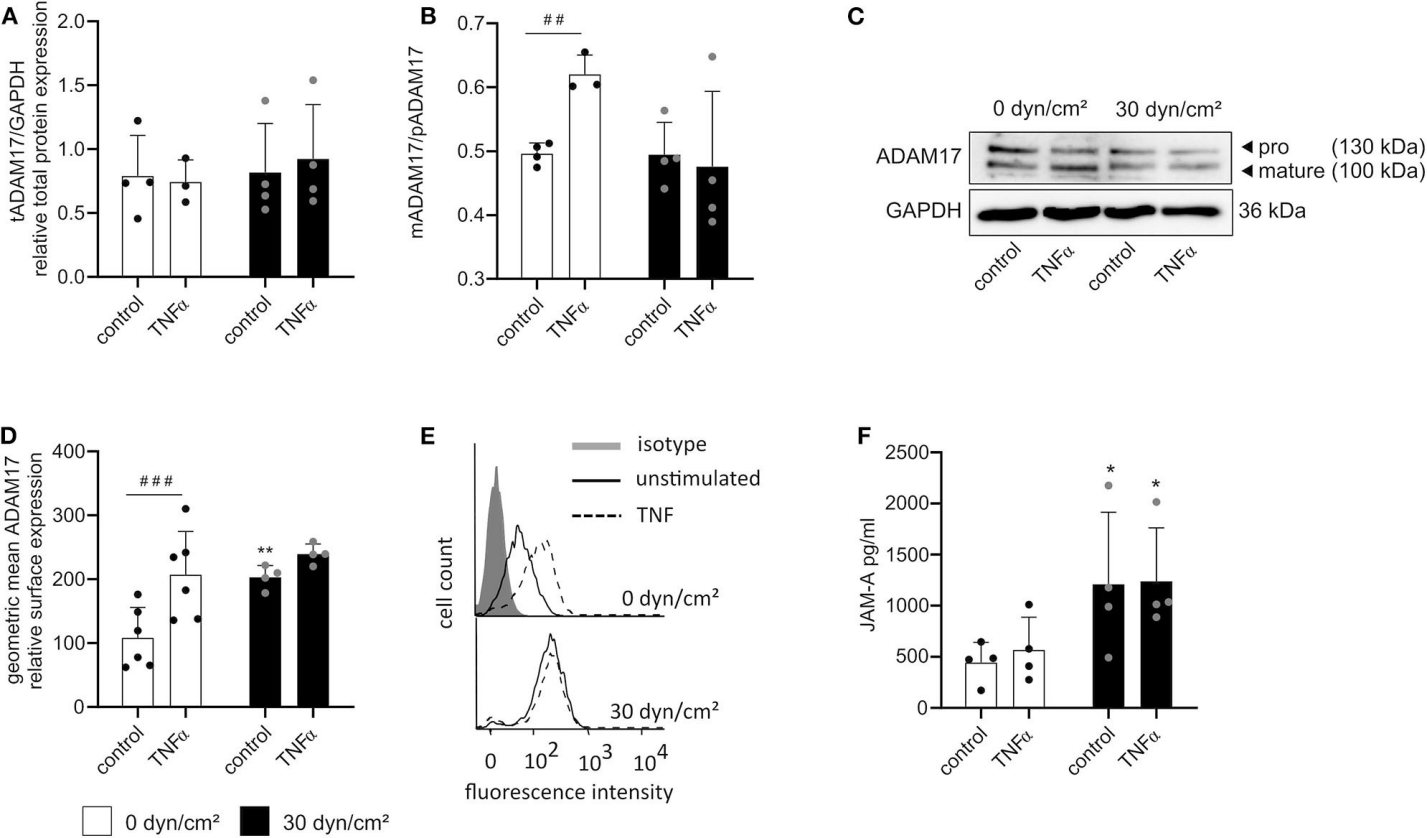
Shear stress- and TNFα-mediated induction of iRhom1 and iRhom2 lead to increased active ADAM17 on the cell surface. HUVECs were cultured for 24 h under static conditions or with a shear stress of 30 dyn/cm^2^ and subsequently stimulated with or without 10 ng/ml TNFα for another 24 h with or without shear stress. Cells were then analyzed for ADAM17 protein expression **(A–C)** and ADAM17 surface expression **(D,E)**. The concentrated supernatant was analyzed for levels of soluble JAM-A **(F)**. **(A–C)** Western blot results are shown as the ratio of the densitometric signal of total ADAM17 (tADAM17) and GAPDH **(A)**, as the ratio of the densitometric signal of mature ADAM17 (mADAM17) and pro ADAM17 (pADAM17) **(B)**, and as representative blot **(C)**. **(D,E)** Results of the flow cytometric analysis are shown as geometric mean of the fluorescence intensity representing the relative ADAM17 surface expression **(D)** and as representative histogram **(E)**. **(F)** Results of the JAM-A ELISA are presented as concentration of soluble JAM-A in pg/ml. Four independent experiments were performed with HUVECs from four different donors. Data are shown as mean + standard deviation (SD) and as black and gray dots representing the individual data points. Statistical differences to the corresponding static control are indicated by asterisks (*p < 0.05, **p < 0.01, and ***p < 0.001) and significant differences between untreated control cells and cells treated with TNFα are indicated as hashes (^#^*p* < 0.05, ^##^*p* < 0.01, and ^###^*p* < 0.001).

Finally, we sought to investigate the influence of both treatments on ADAM17 maturation. HUVECs were cultured as described above and subsequently lysed for Western blotting or harvested from the flow chambers for flow cytometric analysis. Surprisingly, no effect of TNFα stimulation or shear stress on the total protein expression of ADAM17 could be found despite the transcriptional induction which was described above (Figures 8A,C). Consistent with the upregulation of iRhom2 and its reported effects on ADAM17 maturation, an increased presence of the mature form in relation to the proform of ADAM17 was seen in response to stimulation with TNFα alone (Figures 8B,C). This was accompanied by increased surface expression of the protease as demonstrated by flow cytometry (Figures 8D,E). Interestingly, under these conditions shedding of the ADAM17 substrate JAM-A was not increased.

Some of these responses were different for flow exposure. Here we did not observe increased ADAM17 maturation (Figures 8A–C), but nevertheless the surface expression of the protease was upregulated (Figures 8D,E) and this correlated with increased shedding of JAM-A (Figure 8F). A very similar response was obtained when a combination of flow and TNFα was applied. Compared to flow exposure alone, there was no enhancement of maturation (Figures 8A–C), a slight additional increase in ADAM17 surface expression (Figures 8D,E) but no further increase in JAM-A shedding (Figure 8F) when cells were additionally stimulated with TNFα. Noteworthy, JAM-A mRNA expression was neither affected by shear stress nor by TNFα (Supplementary Figure 9).

**Figure 9:**
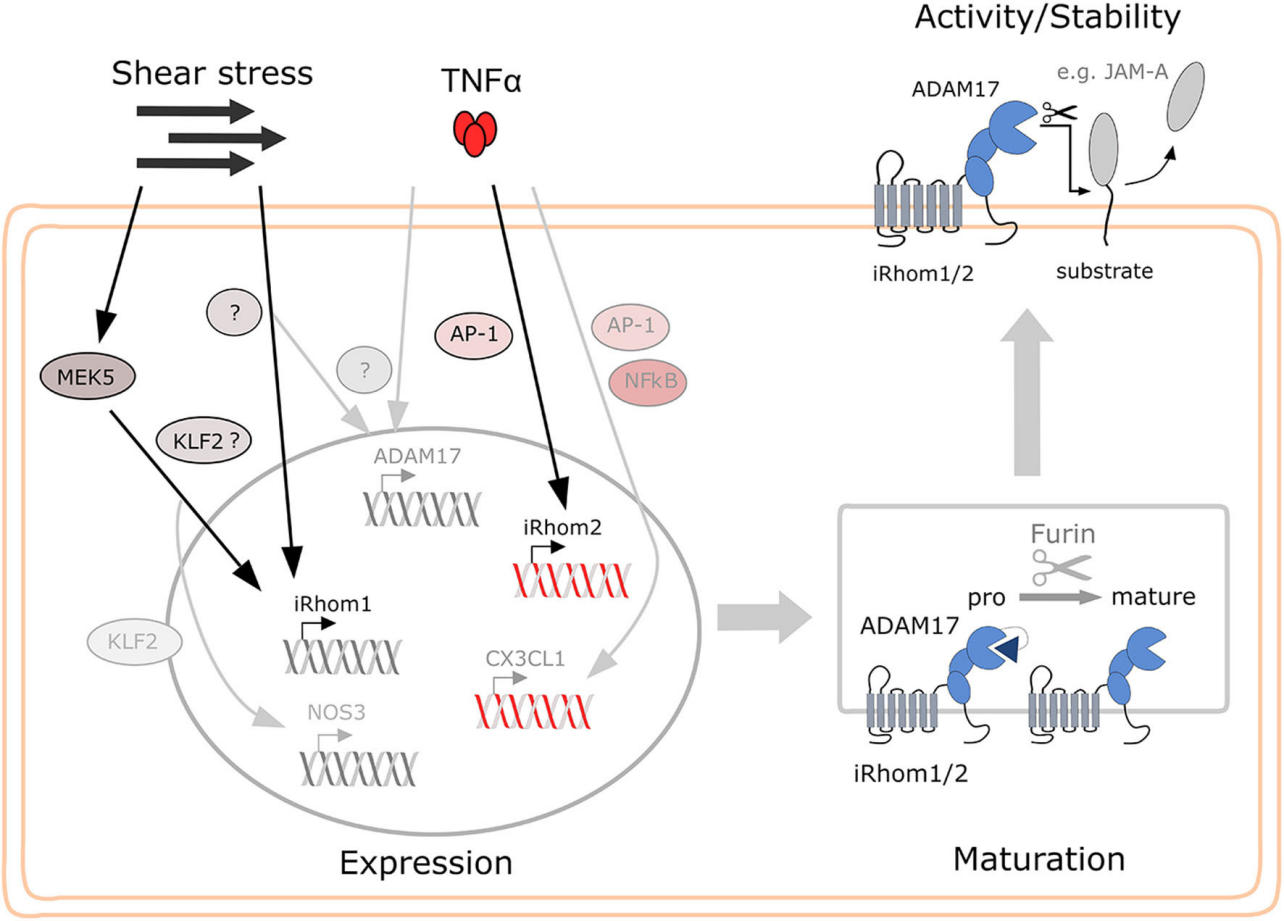
Proposed model for the regulation of iRhom1 and iRhom2 in endothelial by shear stress and TNFα and the consequences for ADAM17 activity on the cell surface. While shear stress mainly induces iRhom1 mRNA expression, which is partially controlled via MEK5 and KLF2, TNFα exclusively induces iRhom2 mRNA expression in endothelial cells predominantly via AP-1. Both iRhoms contribute to increased ADAM17 maturation and surface expression or may even stabilize mature ADAM17 on the cell surface. This can then lead to increased shedding of ADAM17 substrates, such as JAM-A depending on the stimulatory condition.

Overall, it can be noted that flow cultivation is a potent inducer of iRhom1 expression, increased ADAM17 surface expression and JAM-A shedding. Furthermore, induction of iRhom2 by TNFα is correlated with an increase in ADAM17 surface expression but does not lead to enhanced JAM-A shedding (Figure 9).

## Discussion

The importance of iRhom1 and iRhom2 for ADAM17 maturation, surface expression and shedding activity has been convincingly demonstrated in mice with targeted disruption of either iRhom gene. For example, experiments with iRhom2 knockout mice revealed that ADAM17 maturation is completely prevented in hematopoietic cells while it remained unchanged in other tissues (18, 19). Other studies with iRhom1 knockout mice indicated that ADAM17 maturation in non-hematopoietic cell types is regulated by iRhom1 (20, 21). In fact, one of the two initially described iRhom1 knockout mice lines was lethal (20), similar to the ADAM17 knockout (22). Due to the negative effect of iRhom2 knockout on the maturation of ADAM17 in immune cells, the respective mice were mainly studied in animal models of inflammatory diseases, where the iRhom2 knockout often showed a protective effect (8, 19, 23). This was explained by the absent production of soluble TNFα, which is mainly produced by mature ADAM17 on monocytic cells.

Our bioinformatic analyses confirmed that immune cells express considerably more iRhom2 than iRhom1, whereas in endothelial cells iRhom1 is clearly more expressed than iRhom2. Consistent with this, our findings indicate that endothelial cells under conditions of physiologic flow and in the absence of inflammation predominantly express iRhom1. In this situation, iRhom1 might contribute to endothelial homeostasis by being upregulated upon stimulation with physiological shear stress and by promoting increased ADAM17 surface expression. This increased ADAM17 surface expression could be important for the adaptation of endothelial cells to flow conditions. For example, increased JAM-A shedding could contribute to the dissolution of cell-cell contacts and thus enable the typical reorientation of the cells in the direction of flow (22).

However, the role of iRhom1 and iRhom2 in ADAM17 maturation cannot be simply limited to their differential expression in different cell types, such as endothelial cells and leukocytes, respectively. We showed that also iRhom2 can be induced in endothelial cells by the pro-inflammatory cytokines TNFα and IFNγ. The treatment of endothelial cells with TNFα alone already contributes to an enhanced maturation of ADAM17. Thus, surface expression of endothelial ADAM17 is increased during inflammatory progression and can shed inflammatory mediators, such as the membrane-bound chemokine CX3CL1, which is also induced by TNFα treatment. That would promote the recruitment of immune cells to the endothelium and facilitate the inflammatory progression (10). In addition, VCAM-1 shedding can also promote the extravasation of endothelial cells (24). One reason why an inflammatory reaction in the endothelium does not occur immediately under flow conditions may be that some inflammatory ADAM17 substrates, such as VCAM-1 and CX3CL1, are inhibited by physiological shear stress in such a way that even additional TNFα stimulation is not sufficient to induce expression (25, 26). However, if the otherwise rather high physiological shear stress is reduced, the expression of VCAM-1 and CX3CL1 is induced (27–29), which promotes the formation of atherosclerotic plaques (30). Here, the transcriptional induction of both iRhoms could significantly enhance the surface expression of ADAM17 and thus promote vascular permeability and immune cell recruitment through the shedding of VCAM-1 and CX3CL1. This increased ADAM17 maturation would also be expected to increase JAM-A shedding, as other studies have shown that TNFα induces JAM-A shedding in endothelial cells (31). In this study, however, no increased shedding of JAM-A could be observed in the course of TNFα treatment. In the study mentioned above, a slightly earlier time point was chosen and a 5-fold higher dose of TNFα was investigated together with IFNγ. This could explain the observed difference, as we could also show that treatment with high concentrations of TNFα and IFNγ can significantly increase the expression of iRhom2 compared to induction by moderate TNFα concentrations alone. However, the increased expression of iRhom1 by shear stress may also contribute to increased TNFR2 shedding and thus desensitize the endothelial cells for TNFα. This has already been described for the endothelial ADAM17 knockout, where the authors unexpectedly found that the endothelial ADAM17 knockout promotes atherogenesis (32). This could also contribute to the overall anti-inflammatory effect of shear stress on endothelial cells. Noteworthy, the surface expression of ADAM17 is not necessarily correlated to ADAM17 activity since ADAM17-mediated shedding has to be induced on the cell surface (33, 34). This would explain why the TNFα-induced increase in surface ADAM17 is not accompanied by enhanced shedding activity. Here, iRhoms also play a vital role in regulating the shedding process on the cell surface (35, 36) and might also affect the substrate selectivity of ADAM17. For iRhom2 an influence on the substrate selectivity of ADAM17 has already been described (18). Moreover, post-translational modification or conformational changes of iRhoms represent further levels of regulation. For example, different post-translational modifications of the cytoplasmic tail or changes in the transmembrane region of iRhom2 can affect ADAM17 (35–37).

In the present study, we observed some induction of ADAM17 mRNA by shear stress which, however, did not correlate with increased total protein expression of ADAM17. Interestingly, no accumulation of mature ADAM17 is visible despite the upregulation of iRhom1 which promotes the conversion of the proform into the mature form. As an explanation, it can be envisaged that shear stress can also increase the turnover of mature ADAM17. More mature ADAM17 could be transported to the surface in response to shear stress and part of this surface pool could subsequently become degraded. In fact, it was already described that the maturation level and the amount of mature ADAM17 do not necessarily correlate with the ADAM17 surface expression as not all mature ADAM17 molecules are usually transported to the cell surface after maturation (34, 38) It was also found that there is increased internalization upon excessive ADAM17 surface expression and/or after increased shedding activity. If not recycled, ADAM17 is degraded via the lysosomal pathway after its internalization (34, 39). Another possibility is the formation of ADAM17-loaded extracellular vesicles in response to an excess of active ADAM17 (40). Furthermore, iRhoms play a crucial role in stabilizing mature ADAM17. That would also explain the increased ADAM17 surface expression, while the total protein expression was not altered (41, 42). It can therefore be assumed that under shear stress more ADAM17 is produced which also undergoes accelerated maturation due to a higher iRhom1 expression. Shear stress also accelerates the transport of mature ADAM17 to the cell surface, where more ADAM17 is stabilized by iRhom1 than under basal conditions. Yet, an excess of mature and active ADAM17 seems to be cleared from the cell most likely by internalization and degradation or extracellular vesicle formation (40). Under shear stress this system seems to be already saturated and further TNFα-dependent induction of iRhom2 does not enhance ADAM17 maturation and hence surface expression.

The transcriptional long-term regulation by shear stress is usually controlled by MEK5 and an enhanced KLF2 expression (12). Although we obtained some evidence for the role of this pathway for iRhom1 induction in response to flow, it is also clear that other mechanisms must be involved. Possibly, the transcription factor Nrf2 plays a role here. It is described that Nrf2 translocation into the cell nucleus induces by shear stress, which is due to enhanced eNOS activity and an associated ROS production (15, 43). Although the expression of eNOS is also increased via shear stress-dependent KLF2 signaling, eNOS is also directly activated by shear stress mediated by AKT (44), which may promote MEK5/KLF2-independent translocation of Nrf2. In addition, also a shear stress-dependent activation of PPARy has been described (45), which may also be responsible for the transcriptional regulation of iRhoms. Furthermore, especially in the case of iRhom2, a compensatory mechanism after ADAM17 activation may exist. Shear stress could directly induce activation of ADAM17 and subsequently activated ADAM17 could become internalized. In order to compensate for the now reduced surface expression, iRhoms and ADAM17 are transcriptionally induced. The transcriptional regulation mediated by TNFα in endothelial cells is mostly mediated by AP-1 or NFκB activation (46). As shown in our present study, with regard to the TNFα-induced expression of iRhom2, AP-1 seems to be the main regulator among these two possible pathways.

Although our bioinformatic analyses show that iRhom1 is also increasingly expressed in whole vessel samples, it would be of great value to confirm our findings in a 3D cell culture model. This would also allow further exciting investigations. For example, ADAM17 was also shown to be involved in the angiogenesis potential of endothelial cells (47). Furthermore, it was shown that endothelial sprouting can be induced by high shear stress in a 3D cell culture model (48). Therefore, it would be very interesting to investigate whether shear stress-mediated iRhom1 induction and subsequent ADAM17 activation might be responsible for the described endothelial sprouting in a 3D cell culture model. Of course, substrate properties, such as stiffness and composition of the extracellular matrix also play an important role in such a setup. Therefore, their influence on iRhom expression and ADAM17 regulation should be also investigated.

Our findings extend the existing model that iRhom1 expression is predominantly relevant in non-hematopoietic cells while iRhom2 is relevant in hematopoietic cells. Both iRhoms can be regulated on the transcriptional level in endothelial cells. While the function of iRhom1 is enforced by flow conditions, also iRhom2 can become relevant in endothelial cells after inflammatory activation. By this, shear stress as well as inflammatory stimulation can lead to substantial regulation of the metalloproteinase ADAM17 in endothelial cells.

## Data Availability Statement

The raw data supporting the conclusions of this article will be made available by the authors, without undue reservation.

## Ethics Statement

The studies involving human participants were reviewed and approved by Ethic Commission of the Medical Faculty RWTH Aachen vote: EK241/18. The patients/participants provided their written informed consent to participate in this study.

## Author Contributions

AB, SF, DR-G, and AG performed the experiments. AB and SD analyzed the data. AB, AL, PM, and SD designed the study. AB and AL wrote the manuscript. All authors revised the manuscript.

## Conflict of Interest

The authors declare that the research was conducted in the absence of any commercial or financial relationships that could be construed as a potential conflict of interest.
