# Supplementary material for: Differential Induction of the ADAM17 Regulators iRhom1 and 2 in Endothelial Cells
Source: Front Cardiovasc Med. 2020 Dec 1;7:610344. doi: 10.3389/fcvm.2020.610344 (PMC7736406; doi:10.3389/fcvm.2020.610344)
Supplement: Supplementary file 1 [file Data_Sheet_1.PDF]

Supplemental table 1: Used RT-qPCR primers and their annealing temperature.

gene	sequence	annealing temperature
ADAM10	forward: GGATTGTGGCTCATTGGTGGGCA<br>reverse: ACTCTCTCGGGGCCGCTGAC	61 °C
ADAM17	forward: GAAGTGCCAGGAGGCGATTA<br>reverse: CGGGCACTCACTGCTATTACC	55 °C
EDN1	forward: CCTAAGACAAACCAGGTCGG<br>reverse: CTTTGCCAGTCAGGAACCA	60 °C
GAPDH	forward: CGGGGCTCTCCAGAACATCATCC<br>reverse: CCAGCCCCAGCGTCAAAGGTG	66 °C
KLF2	forward: AAAGACCACGATCCTCCT<br>reverse: CTTATTTCTCACAAGGCATCAC	59 °C
NOS3	forward: CGAGTGAACGCGACAATCCT<br>reverse: GCTGCAAAGCTCTCTCCATTC	60 °C
RHBDF1	forward: GACAGCCCACATCTCTTCAC<br>reverse: TCCTTGCTCACTCCAAACCA	56 °C
RHBDF2	forward: CGATTGACCTGATCCACC<br>reverse: CAAAGTCTCCGAGCAGTCC	58 °C
TBP	forward: GAGCCAAGAGTGAAGAACAGTC<br>reverse: GCTCCCCACCATATTCTGAATCT	60 °C

Dataset: 431 anatomical parts from data selection: HS_AFFY_U133PLUS_2-0

Showing 2 measure(s) of 2 gene(s) on selection: HS-0

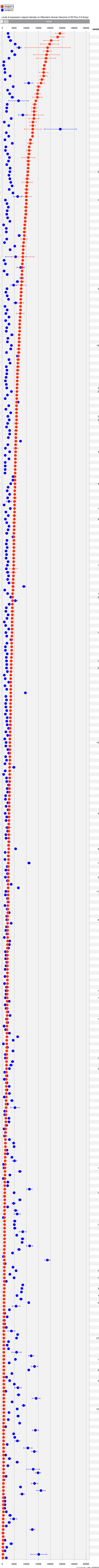

Homo sapiens (431)	samples	avg. expr.
iliac vein endothelium cell	2	24001.35
lung vein endothelium cell	2	23131.66
prostatic stroma cell	6	20356.25
aorta endothelium cell	2	19783.67
coronary artery endothelium cell	2	18947.12
growth plate	9	18828.75
iliac artery endothelium cell	2	18457.47
liver vein endothelium cell	3	18238.93
skeletal muscle derived mesoangioblast	3	18115.01
gingival fibroblast	1	17523.93
synovial membrane fibroblast	7	17299.45
foreskin-derived neonatal fibroblast	2	17211.95
breast (mammary gland) stroma cell	18	17204.33
pulmonary (lung) myofibroblast	12	16477.54
microvessel endothelium cell	3	16124.37
dermal papilla cell	12	15138.04
osteoblast like cell	9	14821.09
dermis	16	14695.41
chorion villus cell	12	14638.02
urinary bladder stromal fibromuscular cell	2	14041.46
umbilical artery endothelium cell	9	13550.03
chondrocyte	2	13306.54
bronchoalveolar derived mesenchymal stromal cell	8	13224.70
basal epidermis cell	3	13223.42
retina microvessel endothelium cell	3	13178.02
meniscal cell	8	12839.55
liver artery endothelium cell	3	12700.65
suprabasal epidermis cell	3	12670.46
chondrogenic progenitor cell	6	12630.37
aortic root wall	38	12619.42
vascular smooth muscle cell	5	11794.73
dermal lymphatic microvessel endothelium cell	10	11670.14
uterine myometrium myocyte	3	11263.73
pulmonary (lung) fibroblast	16	11235.25
placenta	43	11166.89
reticular dermis cell	3	10891.71
trigeminal ganglion	16	10853.61
chorion villus	12	10848.19
buccal epithelium organotypic model	11	10757.77
glomerulus	201	10709.41
lung epithelium cell 3D culture	4	10565.45
iris microvessel endothelium cell	2	10378.59
lung artery endothelium cell	11	10358.18
gingival epithelium organotypic model	12	10218.83
hair follicle keratinocyte	6	10144.45
esophagus (oesophagus)	27	10052.37
conjunctival epithelium cell (sp.)	4	10040.30
dermal fibroblast	124	9988.68
left internal mammary artery	12	9984.58
bone marrow stromal cell	4	9846.10
spinal ganglion (dorsal root ganglion)	16	9837.82
internal mammary artery wall	40	9814.97
sural nerve	35	9558.24
umbilical vein endothelium cell	72	9549.04
bronchial smooth muscle cell	3	9360.01
fetal chondrocyte	11	9226.95
osteoblast	4	9222.61
epidermis	48	9188.80
coronary artery	6	9052.56
uterine endometrium stroma cell	3	8926.66
cardiac microvessel endothelium cell	4	8701.15
bronchial epithelium cell 3D culture	58	8680.65
tarsal conjunctiva	60	8648.56
cerebral cortex astrocyte	2	8567.54
dental pulp stem cell	6	8491.56
coronary artery smooth muscle cell	11	8380.97
nasal epithelium cell	10	8278.36
trabecular meshwork cell	2	8240.37
prostatic stroma fibromuscular cell	5	8204.65
urinary bladder urothelium (epithelium) cell	6	8154.10
tongue squamous epithelium cell	12	8146.69
dermal papilla	3	7973.97
uterine endometrium	320	7906.66
skeletal muscle myoblast	7	7853.85
synovial fluid	1	7750.85
choroid microvessel endothelium cell	3	7683.93
amniocyte	18	7665.38
scalp	36	7600.62
artery	1	7598.74
aorta	4	7504.36
aortic valve cusp (leaflet)	15	7425.96
ovarian granulosa cell	2	7300.38
oral mucosa (unspecified)	87	7258.75
peritoneum	22	7255.27
tumor derived astrocyte	1	7213.24
large airway basal cell	5	7089.08
vagina	8	7078.90
astrocyte (unspecified)	6	7011.16
skin	1998	6979.01
nasopharyngeal (epipharyngeal) epithelium	4	6854.09
uterine cervix	8	6842.81
lung microvessel endothelium cell	3	6714.71
corneal limbus	4	6606.50
penis	6	6494.84
aorta smooth muscle cell	3	6475.11
ovary (female gonad)	9	6392.25
bone marrow derived mesenchymal stem cell	36	6317.79
urethra	8	6313.15
prostate (prostate gland)	22	6235.76
medial rectus muscle	7	6215.27
breast (mammary gland)	323	6156.53
bronchial (primary, main stem) epithelium cell	233	6121.11
minor salivary gland	14	6039.89
nasal organotypic tissue culture	24	6033.23
conjunctival epithelium cell	3	6025.08
choroid	1	5972.79
saphenous vein	6	5957.82
vulva	27	5952.61
colon	241	5895.24
colonic mucosa	208	5894.38
aortic valve	20	5849.81
cornea	6	5847.64
breast (mammary gland) epithelium cell	50	5741.62
pharyngeal mucosa	8	5690.97
trachea	8	5631.56
oral keratinocyte	2	5625.23
nipple	8	5606.09
vein endothelium cell	12	5582.52
lung	472	5526.12
lateral rectus muscle	1	5492.11
conjunctiva	7	5464.41
bronchial organotypic tissue culture	24	5431.02
uterine myometrium	58	5423.80
right ventricle interventricular septum	48	5368.46
mesenchymal stem cell derived adipocyte	6	5365.45
epidermal keratinocyte	13	5303.67
neuron	3	5264.57
nasal epithelium	409	5251.08
fetal retina pigment epithelium cell	12	5246.08
ascending colonic mucosa	35	5229.27
nasopharynx (epipharynx)	6	5184.30
large intestine	6	5180.55
tendon synovium	5	5124.34
umbilical cord	53	5109.48
synovium (synovial membrane)	148	5072.64
heart atrium	8	5069.60
urinary bladder	4	5051.57
subcutaneous adipose tissue (SAT)	453	5021.28
colonic gland (crypt)	10	4956.46
colonic epithelium cell (colonocyte)	40	4926.94
bronchus (primary, main stem)	6	4869.26
joint	3	4867.59
blood outgrowth endothelium cell	4	4866.24
omental adipose tissue	40	4861.13
heart ventricle	6	4857.99
gluteal subcutaneous adipose tissue (glutSAT)	69	4846.51
colorectum	4	4809.95
salivary gland	10	4745.07
skeletal muscle	69	4736.80
adipose tissue (fat)	15	4713.29
sigmoid colonic mucosa	144	4707.21
uterus	2	4697.00
caecum (cecum)	2	4656.47
tongue	19	4655.52
tracheal epithelium cell	44	4542.16
umbilical cord blood derived CD34 hematopoietic stem / progenitor cell	12	4429.58
nurse-like cell	10	4425.90
heart right atrial appendage	19	4412.91
descending colonic mucosa	138	4304.69
gingiva	242	4243.75
reconstructed epidermal model	3	4222.15
mediastinal adipose tissue (MAT, intrathoracic adipose tissue)	64	4193.28
heart	2	4181.81
hepatic myofibroblast	2	4168.83
stomach fundus	8	4164.79
ovarian (surface) epithelium cell	63	4121.27
embryonic cell	6	4117.52
small airway epithelium cell 3D culture	47	4086.96
pyloric antrum	8	4082.15
heart left ventricle	140	4036.10
transverse colon	3	4032.62
liver-infiltrating lymphocyte	9	3932.49
thyroid gland	21	3877.41
parotid gland	30	3877.39
epididymal cauda	3	3873.26
sigmoid colon	30	3854.66
pancreatic islet cell	12	3784.94
rectus abdominus muscle	102	3733.14
heart right ventricle	19	3730.63
subcutaneous abdominal adipose tissue (SAAT)	553	3700.24
stomach cardia	6	3613.13
embryonic prechondrocyte	6	3541.22
heart left atrial appendage	40	3538.16
joint synovium	45	3530.15
seminal vesicle	1	3524.14
renal (kidney) medulla	8	3493.23
macrophage	23	3487.41
biceps brachii	28	3486.39
pancreatic duct (duct of Wirsung)	6	3478.59
labial salivary gland	12	3432.83
fetal retina pigment epithelium	6	3427.95
ovarian stroma cell	8	3416.66
epididymal corpus	3	3406.02
descending colon	6	3343.24
retinal pigment epithelium	2	3307.24
small intestine	3	3293.41
pancreas	70	3266.56
heart muscle (myocardium, cardiac muscle)	38	3235.17
rectosigmoid colonic mucosa	39	3204.39
nasal epithelium cell 3D culture	71	3174.47
vastus lateralis muscle	1021	3169.34
lower lobe bronchiolar epithelium cell	11	3162.68
cumulus cell	37	3161.74
pars reticulata	7	3133.91
duodenum	28	3119.76
epididymal caput	3	3116.03
upper lobe bronchiolar epithelium cell	11	3102.89
lymph node	9	3099.25
deltoid muscle	22	3080.06
embryo	18	3058.03
jejunum	8	3053.41
renal (kidney) cortex	15	3044.02
stomach	46	2986.47
lateral thalamic nucleus	4	2984.24
ascending colon	8	2968.40
quadriceps femoris muscle	37	2966.33
biceps femoris muscle	47	2904.88
gall bladder	1	2870.96
cuboidal alveolar type 2 cell (AT2)	6	2867.36
entorhinal cortex	70	2844.72
bronchiolar (small airway) epithelium cell	596	2818.51
pars compacta	7	2756.86
ileal mucosa	24	2755.46
spinal cord	17	2731.83
pons	2	2721.62
globus pallidus	14	2718.66
cerebral white matter oligodendrocyte progenitor cell	4	2706.77
oviduct (fallopian tube)	3	2697.45
ileal epithelium cell	9	2689.42
medulla oblongata	10	2656.80
hepatocyte	645	2653.57
diaphragm muscle	10	2600.32
subthalamic nucleus	18	2588.48
middle temporal gyrus pyramidal neuron	33	2551.16
monocyte derived macrophage	101	2548.73
basal ganglia	24	2542.32
testicular lobules	45	2528.42
hypothalamus	15	2521.27
renal tubulointerstitium	206	2506.23
ventral tegmental area	16	2459.24
adrenal gland	6	2427.50
tonsil	9	2425.89
kidney	1150	2422.84
Brodmann area 10	51	2408.67
midbrain (mesencephalon)	15	2392.65
orbital adipose tissue	97	2336.80
superior frontal gyrus pyramidal neuron	36	2319.97
superior frontal gyrus	68	2282.25
lacrimal gland	70	2275.60
putamen	27	2270.83
liver	830	2268.93
tonsillar CD8 resting T-cell (unspecified)	5	2234.75
cerebral white matter astrocyte	7	2223.77
posterior superior temporal gyrus (Brodmann area 22)	42	2207.73
postcentral gyrus	66	2200.70
blood erythroid progenitor cell	6	2200.41
adrenal gland cortex (adrenal cortex)	36	2197.83
corpus callosum	18	2178.33
substantia nigra	67	2171.73
retina	36	2171.73
parietal lobe	29	2155.68
entorhinal cortex large stellate neuron	28	2136.12
amygdala	66	2131.39
occipital lobe	18	2122.70
epidermal melanocyte	2	2120.56
superior vestibular nucleus	14	2117.17
testis (male gonad)	10	2117.10
thalamus (dorsal thalamus)	18	2109.37
primary visual cortex pyramidal neuron	34	2108.37
accumbens nucleus	78	2105.84
large airway epithelium cell	176	2092.86
frontal lobe	64	2083.93
rectum	190	2077.83
cerebral white matter	28	2068.08
caudate nucleus	13	2065.76
temporal lobe	17	2061.67
resting T-cell (unspecified)	2	2023.61
tonsillar CD4 resting T-cell (unspecified)	5	2017.39
hippocampus	165	2007.60
brain (encephalon)	54	1992.60
associative striatum	69	1986.07
pituitary gland (hypophysis)	21	1966.83
bone marrow plasma cell	32	1908.93
tonsillar natural killer cell	5	1905.12
spleen	16	1876.08
fetal oligodendrocyte progenitor cell	10	1857.89
cerebral cortex (neopallium)	23	1841.19
vein	1	1837.76
cerebral hemisphere	1	1834.19
prefrontal cortex	101	1810.06
monocyte derived dendritic cell	53	1780.70
tumor derived oligodendrocyte progenitor cell	20	1776.24
pancreatic islet (islet of Langerhans)	248	1725.81
breast (mammary gland) cell	20	1712.55
middle temporal gyrus (Brodmann area 21)	31	1705.89
posterior cingulate gyrus pyramidal neuron	27	1697.32
epicardial adipose tissue (EAT)	46	1694.96
tonsillar CD4 activated T-cell (unspecified)	5	1668.81
transiliac crest core	9	1657.16
tonsillar CD4 regulatory T-cell (unspecified)	5	1653.02
orthochromatophilic normoblast (late erythroblast)	4	1635.53
tonsillar CD8 activated T-cell (unspecified)	11	1577.64
thymus	3	1572.98
dendritic cell	4	1556.38
vermis	1	1534.91
hippocampus pyramidal neuron	28	1499.43
CD4 resting T-cell (unspecified)	1	1498.95
CD8 activated T-cell (unspecified)	1	1447.58
cerebral cortex oligodendrocyte progenitor cell	3	1443.08
frontal pole	3	1403.27
blood eosinophil granulocyte	7	1371.91
cerebellum	49	1339.05
CD8 resting T-cell (unspecified)	1	1317.16
umbilical cord blood neutrophil granulocyte	3	1312.60
oocyte	3	1289.98
bone marrow erythroblast	3	1281.30
diencephalon	1	1235.53
bronchoalveolar system cell	57	1220.74
natural killer cell	3	1197.03
fetal retina	12	1189.88
Brodmann area 46	112	1135.47
peripheral blood NK T-cell (unspecified)	12	1133.22
CD4 activated T-cell (unspecified)	1	1107.12
cardiomyocyte (heart muscle cell)	7	1081.51
genu	32	1030.32
tumor derived CD4 T-cell (unspecified)	26	1028.89
cerebral white matter microglia cell	5	1025.00
peripheral blood CD4 naive T-cell	149	1024.55
polychromatophilic normoblast (intermediate erythroblast)	4	1012.68
bone marrow endothelium progenitor cell	12	1001.78
tonsillar resting T-cell (unspecified)	5	990.27
peripheral blood CD4 activated T-cell (unspecified)	20	964.91
conventional dendritic cell	3	963.08
tonsillar activated T-cell (unspecified)	5	940.36
amniotic fluid	9	930.18
peripheral blood CD8 effector memory RA T-cell	4	921.03
bone marrow	153	908.26
peripheral blood T-cell (unspecified)	2	904.50
plasma cell	3	895.03
synovial fluid mononuclear cell	21	892.71
bone marrow myeloid cell (unspecified)	3	890.50
peripheral blood plasma cell	2	883.16
B-cell	21	880.98
peripheral blood natural killer cell	16	872.82
peripheral blood CD8 resting T-cell (unspecified)	1	870.73
double positive thymocyte	3	861.36
peripheral blood B-cell (unspecified)	59	856.75
Langerhans cell	13	856.42
bone marrow mononuclear cell	19	839.60
peripheral blood leukocyte	176	822.37
peripheral blood conventional dendritic cell	1	817.21
alveolar macrophage	128	814.63
peripheral blood CD4 helper T-cell (unspecified)	4	814.15
peripheral blood plasmacytoid dendritic cell	5	804.85
monocyte	200	796.94
peripheral blood monocyte	99	784.77
peripheral blood granulocyte	117	784.12
tonsillar B-cell (unspecified)	9	770.08
peripheral blood lymphocyte	225	750.01
peripheral blood CD4 T-large granular lymphocyte	3	744.39
inguinal lymph node	94	740.14
CD4 single positive thymocyte	3	706.71
peripheral blood CD4 central memory T-cell	34	705.23
proerythroblast	4	685.70
blood colony-forming unit-erythroid (CFU-E)	3	680.20
platelet (thrombocyte)	28	678.56
bone marrow hematopoietic stem cell	6	673.77
leukocyte	80	670.85
peripheral blood mononuclear cell	2757	664.49
peripheral blood CD4 effector memory T-cell	31	664.12
peripheral blood CD8 naive T-cell	28	662.81
umbilical cord blood cell	3	658.38
T-cell	4	657.84
germinal center B-cell	3	652.39
peripheral blood CD8 T-cell (unspecified)	112	651.13
CD4 regulatory T-cell (unspecified)	30	645.81
lymphocyte	35	644.76
sputum	235	644.08
peripheral blood CD8 central memory T-cell	23	642.52
lymph node CD4 T-cell (unspecified)	10	640.84
peripheral blood CD4 T-cell (unspecified)	204	639.84
pulmonary conventional dendritic cell	27	635.67
tonsillar germinal center B-cell	5	624.15
peripheral blood CD4 regulatory T-cell (unspecified)	27	622.32
tonsillar memory B-cell (unspecified)	6	621.17
memory B-cell	3	614.64
tonsillar plasmablast	7	609.53
blood neutrophil granulocyte	36	608.83
blood	4126	607.10
peripheral blood CD4 resting T-cell (unspecified)	5	606.74
peripheral blood CD8 effector memory T-cell	22	603.75
tonsillar CD8 T-cell (unspecified)	7	602.95
bone marrow monocyte	16	600.22
peripheral blood CD4 stem cell memory T-cell	6	600.07
umbilical cord blood naive B-cell	2	595.72
pulmonary Langerhans cell	7	592.90
peripheral blood CD8 memory T-cell (unspecified)	29	589.53
tonsillar naive B-cell	6	582.40
internal (medial) globus pallidus	18	581.47
plasmacytoid dendritic cell	11	568.28
neutrophil granulocyte	78	553.95
tumor derived T-cell (unspecified)	10	536.69
bone marrow cell	2	535.57
tonsillar centrocyte	15	524.31
tumor derived CD8 T-cell (unspecified)	8	520.51
peripheral blood transitional stage B-cell (unspecified)	4	517.62
peripheral blood memory B-cell (unspecified)	28	517.24
tonsillar CD4 T-cell (unspecified)	7	515.87
bone marrow multipotent progenitor (MPP) cell	1	515.83
naive B-cell	3	508.98
cerebrospinal fluid cell	86	494.38
umbilical cord blood transitional stage B-cell	4	473.67
tonsillar centroblast	15	466.16
peripheral blood CD4 naive regulatory T-cell	9	460.69
tonsillar T-cell (unspecified)	8	434.84
peripheral blood CD4 memory regulatory T-cell	12	429.12
bone marrow granulocyte-monocyte progenitor (GMP) cell	5	426.68
peripheral blood CD4 memory T-cell (unspecified)	50	425.06
thrombus derived leukocyte	4	421.16
bone marrow polymorphonuclear cell (PMN)	3	404.50
band cell	4	390.64
bone marrow CD4 T-cell (unspecified)	7	389.70
peripheral blood naive B-cell	18	387.93
bone marrow common myeloid progenitor (CMP) cell	3	380.28
bone marrow megakaryocyte-erythroid (MEP) progenitor cell	2	365.78
early promyelocyte	3	332.67
metamyelocyte	3	308.92
myelocyte	2	308.24
late promyelocyte	3	291.28
peripheral blood anergic B-cell	5	242.55
peripheral blood CD8 activated T-cell (unspecified)	5	237.71

created with GENEVESTIGATOR

Supplemental Figure 1: Bioinformatic analysis of public transcriptomic data.

Transcriptome data from human samples generated with Affymetrix Human Genome U133Plus 2.0 Arrays from different public were analyzed using the Genevestigator suite (9). Expression patterns of iRhom1 (red) and iRhom2 (blue) mRNA in the data sets of untreated, mock or placebo treated samples of human origin are presented and samples from vascular origin were highlighted in yellow.

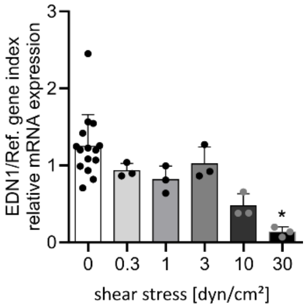

Supplemental Figure 2: Shear stress reduces endothelin-1 mRNA expression.

HUVECs were cultured for 24 h under different flow conditions resulting in the indicated levels of laminar shear stress. Cells were then analyzed for mRNA expression of Endothelin-1 (EDN1) in relation to a reference gene index consisting of GAPDH and TBP. The three independent experiments were performed with HUVECs from three different donors. A static control (0 dyn/cm²) was made for each shear stress level. All static controls were merged in one column for clarity. Data are shown as mean + standard deviation (SD) and as black and grey dots representing the individual data points. Statistical differences to the static control are indicated by asterisks (* $p < 0.05$, ** $p < 0.01$ and *** $p < 0.001$).

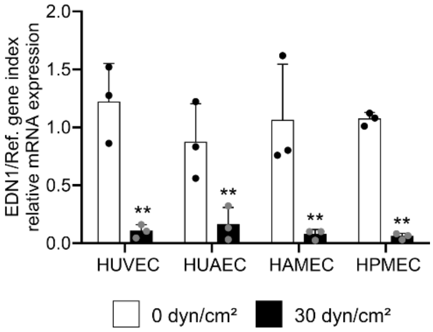

Supplemental Figure 3: High physiological shear stress reduces endothelin-1 mRNA expression in endothelial cells from different vascular beds.

HUVECs, HUAECs, HAMECs and HPMECs were cultured for 24 h with a laminar shear stress of 30 dyn/cm². Cells were then analyzed for mRNA expression of EDN1 in relation to a reference gene index consisting of GAPDH and TBP. The three independent experiments were performed with HUVECs and HUAECs from three different donors and HAMECs and HPMECs from two different donors. Data are shown as mean + standard deviation (SD) and as black and grey dots representing the individual data points. Statistical differences to the static control are indicated by asterisks (* $p < 0.05$, ** $p < 0.01$ and *** $p < 0.001$).

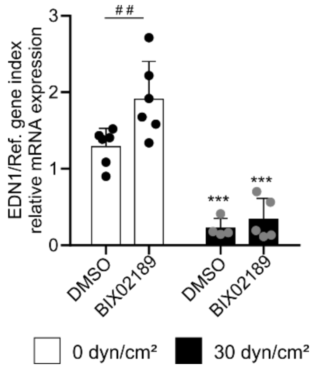

Supplemental Figure 4: Shear stress-mediated reduction of endothelin-1 mRNA expression is not influenced by MEK5 inhibition.

HUVECs were pretreated with DMSO or 10 µM of the MEK5 inhibitor BIX02189 for 2.5 h and cultured for 24 h with a laminar shear stress of 30 dyn/cm² in the presence of DMSO or BIX02189. Cells were then analyzed for mRNA expression of EDN1 in relation to a reference gene index consisting of GAPDH and TBP. At least four independent experiments were performed with HUVECs from four different donors. Data are shown as mean + standard deviation (SD) together with the independent data points as black and grey dots. Statistical differences to the corresponding static control are indicated by asterisks (* $p < 0.05$, ** $p < 0.01$ and *** $p < 0.001$) and significant differences between DMSO and the MEK5 inhibitor are indicated as hashes (# $p < 0.05$, ## $p < 0.01$ and ### $p < 0.001$).

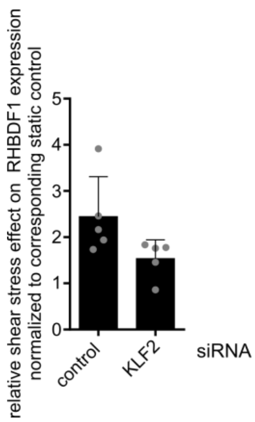

Supplemental Figure 5: Shear stress-mediated induction of iRhom1 mRNA expression could depend on the induction of the transcription factor KLF2.

HUVECs were transfected with non-targeting control siRNA or siRNA against KLF2. After 24 h, cells were cultured under static conditions or with a shear stress of 30 dyn/cm² for 24 h and analyzed for their mRNA expression of RHBDF1. Five independent experiments were performed with HUVECs from five different donors. Data are normalized to the corresponding 0 dyn/cm² control and shown as mean relative shear stress effect + standard deviation (SD) and as black dots representing the individual data points.

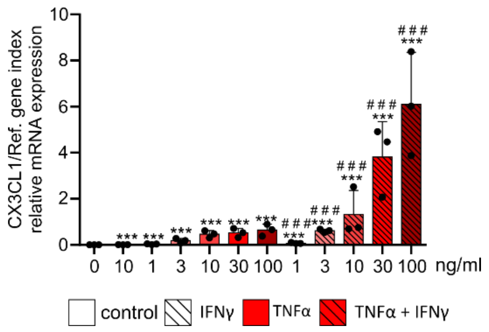

Supplemental Figure 6: Inflammatory cytokines induce CX3CL1 mRNA expression in a synergistic manner.

HUVECs were treated for 24 h with the indicated concentrations of TNFα, IFNγ or the combination of TNFα and IFNγ. Cells were then analyzed for mRNA expression of CX3CL1. Three independent experiments were performed with HUVECs from three different donors. Data are shown as mean + standard deviation (SD) and as black and grey dots representing the individual data points. Statistical differences to the untreated control cells are indicated by asterisks (* $p < 0.05$, ** $p < 0.01$ and *** $p < 0.001$) and significant differences between TNFα and IFNγ treated cells to the corresponding cells treated with TNFα alone are indicated as hashes (# $p < 0.05$, ## $p < 0.01$ and ### $p < 0.001$).

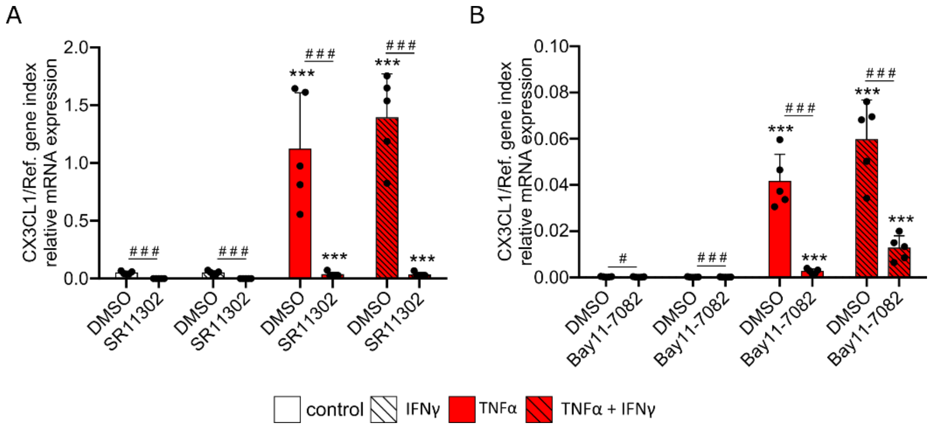

Supplemental Figure 7: Cytokine-induced CX3CL1 mRNA expression in endothelial cells is suppressed by AP-1 and NFκB inhibitors.

HUVECs were pretreated with DMSO, 10 µM of the AP-1 inhibitor SR11302 (A) or 3 µM of the NFκB inhibitor Bay11-7082 (B) for 1 h and cultured for 24 h with 10 ng/ml TNFα, IFNγ or the combination of TNFα and IFNγ in the presence of DMSO, SR11302 or Bay11-7082. Cells were then analyzed for mRNA expression of CX3CL1. Five independent experiments were performed with HUVECs from five different donors. Data are shown as mean + standard deviation (SD) and as black and grey dots representing the individual data points. Statistical differences to the corresponding untreated control cells are indicated by asterisks (* $p < 0.05$, ** $p < 0.01$ and *** $p < 0.001$) and significant differences between DMSO and SR11302 or Bay11-7082 are indicated as hashes (# $p < 0.05$, ## $p < 0.01$ and ### $p < 0.001$).

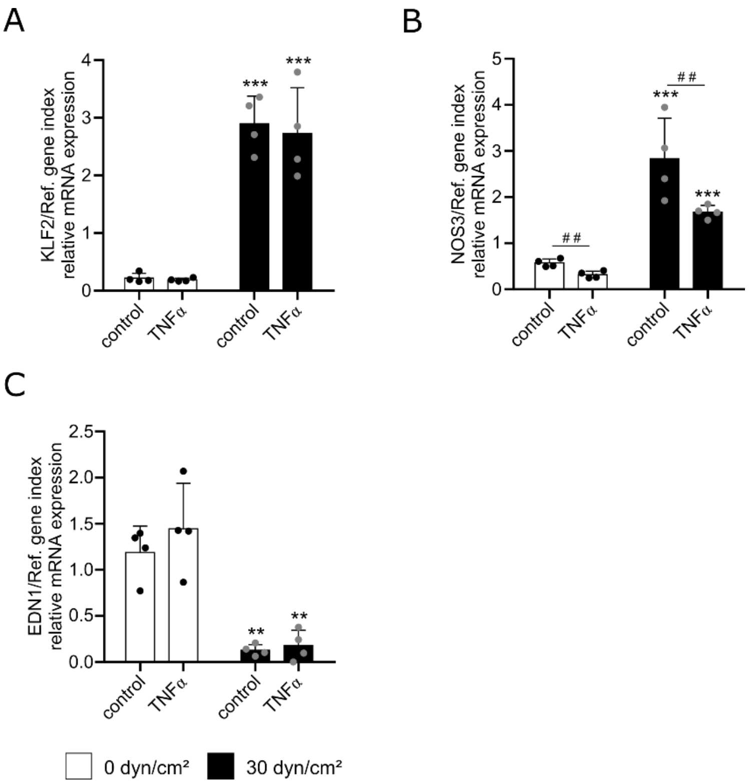

Supplemental Figure 8: Shear stress-induced NOS3 mRNA expression is reduced by additional TNFα treatment.

HUVECs were cultured for 24 h under static conditions or with a shear stress of 30 dyn/cm² and subsequently stimulated with or without 10 ng/ml TNFα for another 24 h with or without flow. Cells were then analyzed for mRNA expression of KFL2 (A), NOS3 (B) and EDN1 (C). Four independent experiments were performed with HUVECs from four different donors. Data are shown as mean + standard deviation (SD) and as black and grey dots representing the individual data points. Statistical differences to the corresponding static control are indicated by asterisks (* $p < 0.05$, ** $p < 0.01$ and *** $p < 0.001$) and significant differences between untreated control cells and cells treated with TNFα are indicated as hashes (# $p < 0.05$, ## $p < 0.01$ and ### $p < 0.001$).

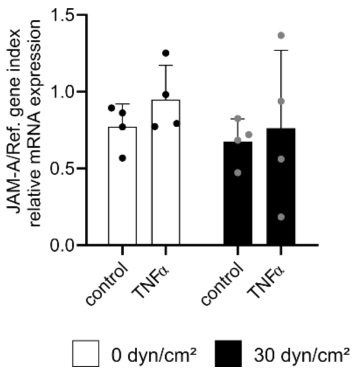

Supplemental Figure 9: JAM-A mRNA expression is neither regulated by shear stress nor by TNFα. HUVECs were cultured for 24 h under static conditions or with a shear stress of 30 dyn/cm² and subsequently stimulated with or without 10 ng/ml TNFα for another 24 h with or without flow. Cells were then analyzed for mRNA expression of JAM-A. Four independent experiments were performed with HUVECs from four different donors. Data are shown as mean + standard deviation (SD) and as black and grey dots representing the individual data points.
